# A novel transgenic reporter of extracellular acidification in zebrafish elucidates skeletal muscle T‐tubule pH regulation

**DOI:** 10.1002/dvdy.770

**Published:** 2025-01-22

**Authors:** Leif R. Neitzel, Maya Silver, Aaron H. Wasserman, Samantha Rea, Charles C. Hong, Charles H. Williams

**Affiliations:** ^1^ Department of Medicine Michigan State University College of Human Medicine East Lansing Michigan USA; ^2^ Henry Ford Health + Michigan State Health Sciences Detroit Michigan USA; ^3^ Department of Medicine University of Maryland School of Medicine Baltimore Maryland USA

**Keywords:** acid homeostasis, development, myotome, transverse tubules, T‐tubules

## Abstract

Disruption of extracellular pH and proton‐sensing can profoundly impact cellular and protein functions, leading to developmental defects. To visualize changes in extracellular pH in the developing embryo, we generated a zebrafish transgenic line that ubiquitously expresses the ratiometric pH‐sensitive fluorescent protein pHluorin2, tethered to the extracellular face of the plasma membrane using a glycosylphosphatidylinositol (GPI) anchor. Monitoring of pHluorin2 with ratiometric fluorescence revealed dynamic and discrete domains of extracellular acidification over the first 72 h of embryonic development. These included acidification of the notochord intercalations, transient acidification of the otic placode, and persistent acidification of the extracellular space of the myotome at distinctly different pH from that within the T‐tubules. Knockdown of centronuclear myopathy genes *Bin1b* (OMIM: 255200) and *MTM1* (OMIM: 310400), which disrupt T‐tubule formation, also disrupted myotome acidification. In this study we visualize extracellular acidic microdomains in the tissues of whole live animals. This real‐time reporter line for directly measuring changes in extracellular pH can be used to illuminate the role of extracellular pH in normal physiological development and disease states.

## Key Findings

- Novel transgenic zebrafish model: Tg(ubi:pHluorin2‐GPI) Embryo extracellular acidification (24‐72 hpf) Spatiotemporal patterns of extracellular acidification in notochord, otic placode, and skeletal muscle Myocyte extracellular acidification in early development and under two models of centronuclear myopathy T‐tubule specific acidification is different than the intermyocyte space

## 1 INTRODUCTION

Extracellular protons (H^+^) are gaining recognition as critical players in cell‐to‐cell communication across diverse contexts. Pioneering work in *Caenorhabditis elegans* revealed that waves of proton flux are essential for proper intestinal muscle function.^1^ This finding has since been echoed in mammals, where proton‐sensing G protein‐coupled receptors (GPCRs) have been implicated in various diseases, including inflammatory bowel disease, asthma, vascular permeability, and cancer progression in mice.^2^, ^3^, ^4^, ^5^, ^6^, ^7^, ^8^, ^9^, ^10^ Furthermore, mutation of SLC9A1, encoding a major Na^+^/H^+^ exchanger, causes ataxia–deafness Lichtenstein–Knorr syndrome.^11^ It is clear that proper regulation of extracellular pH plays a crucial role in health.

However, the role of extracellular protons during development remains less understood, but several lines of evidence suggest proper pH regulation is essential for proper embryonic development. Studies in *Xenopus laevis* (African clawed frog) demonstrated that V‐ATPase, a protein complex that pumps protons out of cells, is essential for proper craniofacial development.^12^ Similarly, zebrafish with mutations in atp6v1e1b, encoding a V‐ATPase subunit, develop malformations mirroring human ARCL type 2C syndrome, a condition linked to dysfunctional pH regulation.^13^ Further supporting this idea are phenotypes emerge with mutations in a zebrafish transcription factor gene, gcm2, crucial for specialized cells that regulate pH in fish called ionocytes. Loss of gcm2 impairs ionocyte activity leading to hair cell malformation in the inner ear, and defects in craniofacial development.^14^, ^15^ Finally, recent research using both chemical and genetic approaches in zebrafish shows that GPR68, a proton‐sensing GPCR, plays a critical role in cranial cartilage and notochord development.^16^ These findings highlight the importance of proper extracellular pH regulation during development.

The measurement of extracellular protons for prolonged periods in vivo is hindered by two technical challenges. First, in contrast to many other cations (e.g., Ca^2+^ and Fe^2+^), protons do not bind to proteins and can therefore disperse significantly faster. Thus, rapid diffusion reduces the accuracy of probe‐based measures.^17^, ^18^ Secondly, fluorescent dyes such as carboxy‐SNARFs and BCECFs, which measure pH through changes in spectral shape upon proton binding, suffer from rapid photo‐bleaching and long‐term toxicity. To overcome these limitations, we developed a novel transgenic zebrafish line, Tg(ubi:pHluorin2‐GPI). The pHluorin2 protein is a ratiometric fluorescent pH sensor specifically designed for the normalization of signal‐to‐protein expression levels.^19^ In ubiquitously expressing pHluorin2 tethered to the outer surface of cell membranes using a glycosylphosphatidylinositol (GPI) signal sequence, this transgenic line expresses pHluorin2‐GPI on the extracellular face of the plasma membrane of every cell in the body. Using this model, we observed dynamic changes in extracellular pH in the notochord, otic placode, and muscle tissues. Furthermore, within the skeletal muscles, we observed unique differences in acidification of the specialized T‐tubules vs. the rest of the myocyte. Notably, we observed tight regulation of proton burden within muscle T‐tubules, even when the surrounding space (inter‐myocyte interstitium) experienced a heavier proton load. Through utilization of our novel Tg(ubi:pHluorin2‐GPI) model, we demonstrate the first evidence of dynamic changes in proton burden occurring within these distinct tissue compartments during development.

## 2 RESULTS

### 2.1 Generation of a transgenic extracellular proton reporter fish

**FIGURE 1:**
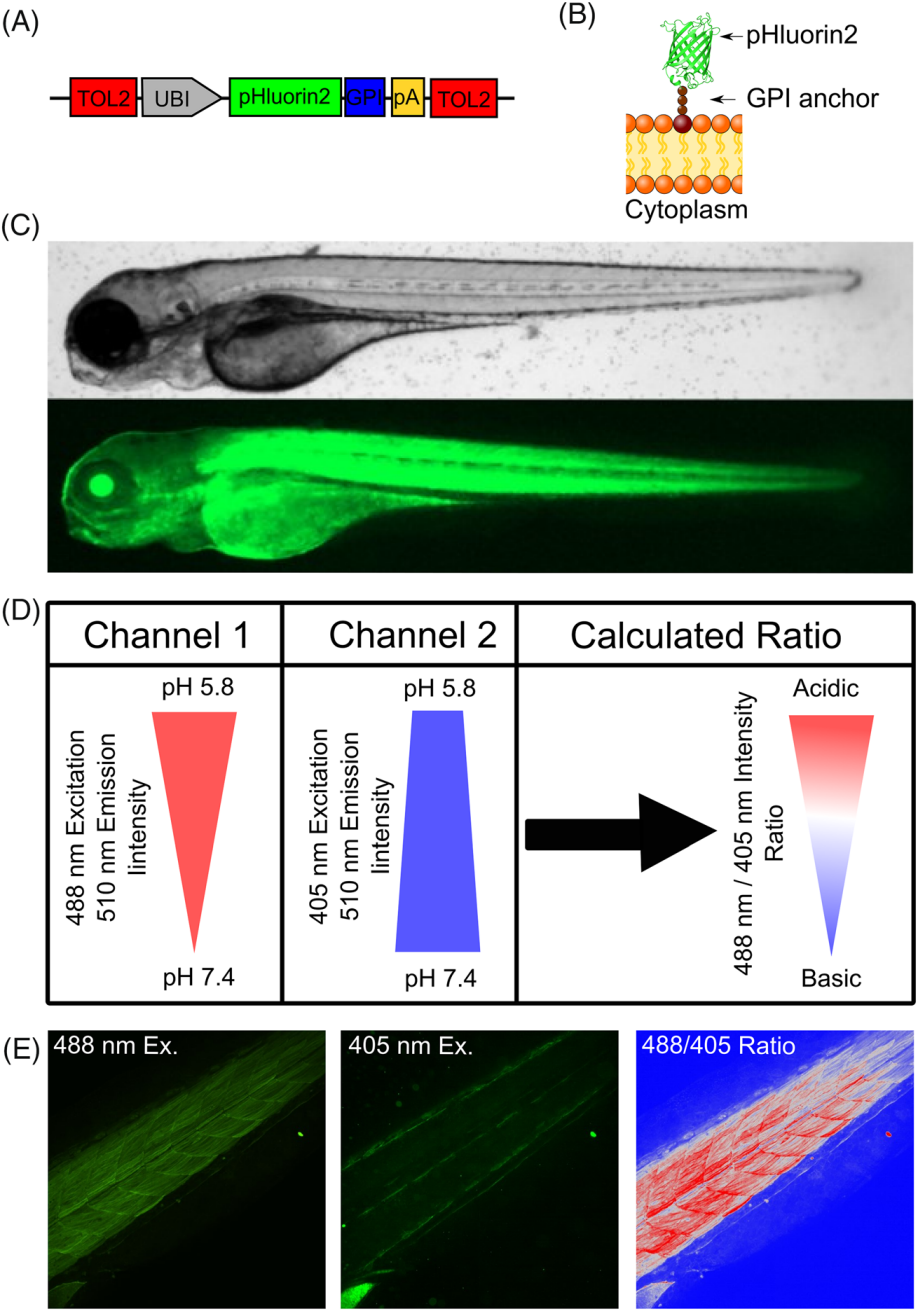
Generation of a ubiquitous pH‐reporting zebrafish line. (A) A zebrafish ubiquitin promoter was placed upstream of a pHluorin2 with a C‐terminal fused to a GPI anchor with a short linker sequence, followed by a poly A tail. This cassette was flanked by Tol2 integration sequences. (B) Schematic of resulting protein expression. The ratiometric pH sensor pHluorin2 localizes exclusively to the extracellular space on the cell membrane. (C) Sagittal brightfield and GFP channel images of a 2 dpf Tg(phlourin2‐GPI) zebrafish. (D) The mathematical paradigm used in this article for ratiometric calculations. (E) Examples of sagittal images of the distal tail from a 2 dpf embryo showing excitation at 488 and 405, as well as the pseudo‐color representation used in this article. (C) 2.5× magnification.

To study extracellular protons in vertebrate development and disease, we choose zebrafish for their optical transparency, rapid development, and tractability to chemical and genetic manipulations. Next, we attached a GPI anchor signal sequence to the C‐terminus of pHluorin2 and placed it under the Zebrafish ubiquitin (ubi) promoter with a Simian virus 40 late polyadenylation signal (Figure 1A; Supplemental File 1). The cassette was flanked by the Tol2 ITRs for Tol2 transposase mediated genomic integration to increase rates of transgenesis.^20^, ^21^ The pH‐sensitive GFP variant, pHlourin2, is a dual excitation fluorophore with emission at 510 nm. The two excitation peaks are at 488 nm, which increases emission significantly with decreasing pH, the second peak at 405 nm is “pH insensitive,” while not increasing emission with decreasing pH, only slightly decreases emission intensity.^19^ By taking the quotient of the pH‐sensitive fluorescence (Ex = 488 nm) divided by pH‐insensitive fluorescence (Ex = 405 nm) we minimize errors due to uneven distribution of the fluorophore.^19^ The GPI anchor targets and tethers pHluorin2 to the extracellular face of the cell membrane. This GPI anchoring strategy has previously been used by Stawicki et al. to show extracellular proton changes in zebrafish inner ear hair cells.^14^ The GPI anchor tether is 1–2 nm long, and pHluorin2 is approximately the size of GFP, which is 3–4 nm, for a total distance of 4–7 nm from the plasma membrane (Figure 1B).^22^, ^23^ Being anchored to the plasma membrane via GPI, the phluorin2 probe offers excellent spatial resolution for measuring pH in the extracellular space most proximal to the cell membrane. However, unlike previously published usage which expressed the pHluorin2‐GPI on a myo6b promoter to label hair cells,^14^ this line was developed to unbiasedly investigate extracellular pH throughout the entire embryo by using a ubiquitous promoter.

We used Tol2 transposon‐based transgenesis to incorporate our pHluorin2‐GPI cassette into the zebrafish genome.^19^, ^20^, ^21^ The construct was co‐injected with mRNA encoding tol2 transposase into Zebrafish embryos at the 1–2 cell stage.^20^, ^21^ All injected embryos were raised until adulthood, out‐crossed with wild‐type (WT) animals, and the offspring assessed for transgene expression. We identified a single male F0 founder with incorporation of the transgene into the gametes. Our F0 founder as well as our F1 and F2 progeny were outcrossed with WTs. Adult fish retain transgene activity and were screened using keratocyte explants (Figure S1). Alternatively, their progeny was screened at 5 days postfertilization (dpf) with a fluorescent stereomicroscope for 488 nm fluorescence (Figure 1C). The F3 was then in‐crossed to homozygosity to control for variability in gene expression levels between embryos within the same clutch. All studies herein were performed with F4 or later homozygotes. Outcrossing the homozygous line to WTs produced heterozygous offspring that reliably expressed half as much pHluorin2‐GPI (Figure S2).

A ratio of 488 nm signal to 405 nm signal was used to determine pH shifts for this article (Figure 1D,E). Immersion in low pH E3 media or treatment with an ionophore, nigericin, to disrupt ionocyte function and embryo pH regulation resulted in higher 488/405 nm ratios verifying functionality of the fluorophore in the model system (Figure S3). The transgene signal was retained upon fixation with 4% paraformaldehyde however we observed an increase in both the 488 nm and 405 nm channels suggesting a change in fluorophore fidelity (Figure S4). Therefore, all other experiments are preformed using live specimens. We developed and validated a novel homozygous transgenic zebrafish line for a ubiquitously expressed, ratiometric, extracellular pH reporter named Tg(Ubi:pHluorin2‐GPI).

### 2.2 Extracellular acidification during notochord formation

**FIGURE 2:**
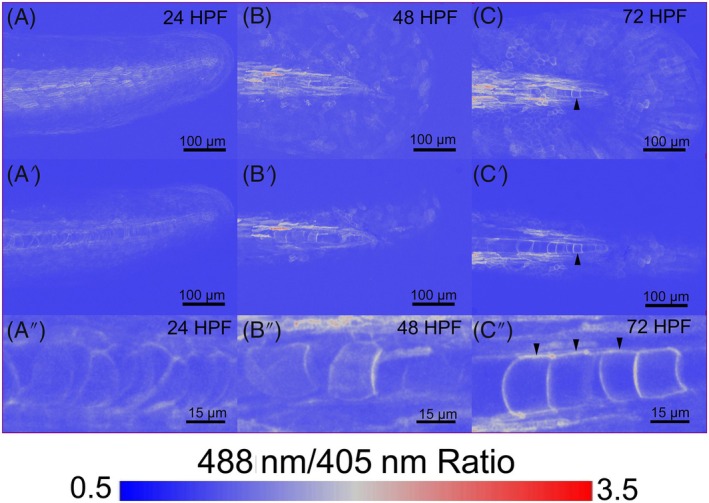
Acidic domains in the notochord during early development. Sagittal images of the distal tail. (A) At 24 hpf the zebrafish tail exhibits limited acidification. Z‐projection of all slices. (A′) A single representative Z‐slice through the tail. (A″) Inset of (A′) showing intercalations of cells and the notochord. (B), (B′), and (B″) Images at 48 hpf show myocyte acidification, with limited acidification in the notochord. (C), (C′), and (C″) Images at 72 hpf show more pronounced acidification in myocytes, very mild acidification observed in epithelia of the tail, and acidified puncta along the notochord lamina (arrowhead). (A–C) Representative images from *n* ≥ 3 biological repeats; 20× magnification.

Notochord formation in zebrafish embryos relies on the interplay between cellular structures and physical forces. Approximately 24 hours postfertilization (hpf), the notochord emerges within the ventral mesoderm as a solid rod. The notochord is composed of an extracellular matrix forming a sheath around cells with large fluid filled vacuoles and is essential for elongation along the body axis.^24^ These fluid‐filled sections act like pressurized balloons, inflating the notochord and enabling its growth while maintaining its shape. Formation of this structure requires the activity of H^+^‐ATPase and mutations in this protein result in undulating notochords.^25^, ^26^ Previous studies using Lysotracker (an acidic organelle stain) failed to detect acidified vacuoles at 24 h. We sought to determine if the extracellular space was acidifying instead. A gradual increase in extracellular proton concentrations between 24‐ and 72‐hpf was observed in the notochord (Figures 2 and S5A,B). Notably the most intense extracellular acidification appears to be at distinct foci along the lamina of the notochord. These data suggest a role extracellular acidification may play a role in the maintenance and growth of the notochord early in development.

### 2.3 Transient extracellular acidification of otic placode

**FIGURE 3:**
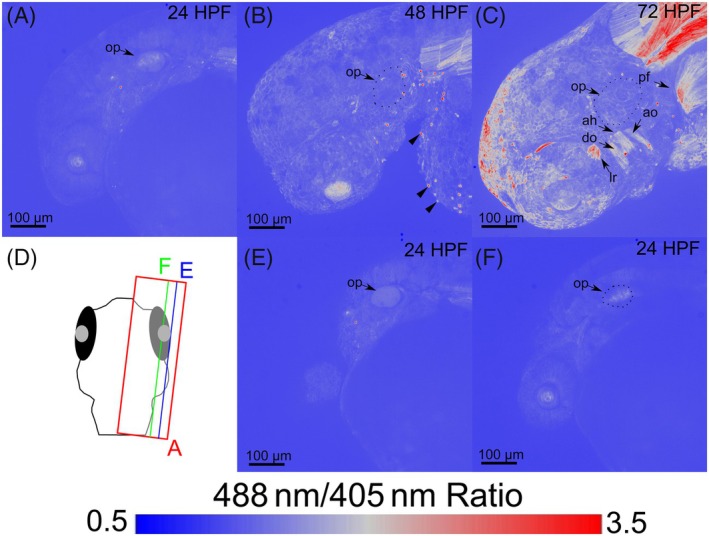
Acidic domains in the larval zebrafish head. (A) At 24 hpf an acidic signal is observed in the otic placode (op), with little acidification across the rest of the larval head. (B) Forty‐eight hours postfertilization the acidification of the otic placode dissipates, and a strong signal appears in the lens. (C) At 72 hpf, skeletal muscle groups of the head and trunk acidify significantly. (D) Schematic representation of the full Z‐stack in (A) and single Z‐stack slices depicted in (E) and (F). (E) and (F) The observed signal in (A) of the otic placode can be separated into two signals. A smooth and broad signal in (E) and a more discrete signal in (F). (A–C) Representative images from *n* ≥ 3 biological repeats; 20× magnification. (Arrows: muscles are designated following the scheme of Schilling and Kimmel 1997: ah, Abductor hyomandibulae; ao, Abductor opercula; do, Dilator opercula; lr, Lateral rectus; op, Otic placode; pf, pectoral fin. Ionocytes. Arrowhead: Ionocytes).

Extracellular acidification has previously been shown to play a critical role in inner ear development. In mice, mutations in a specific subunit of the H^+^‐ATPase protein (H^+^‐ATPase a4) lead to severe hearing loss and enlarged inner ear compartments.^27^ Similarly, human patients with a condition called distal renal tubular acidosis (dRTA) often experience sensorineural hearing loss. This condition is caused by mutations in genes encoding subunits of the H^+^‐ATPase transporter loss.^13^, ^28^, ^29^, ^30^, ^31^ Lastly, zebrafish mutants lacking a specific transcription factor (gcm2) provide further evidence for the link between proton balance and inner ear health. These mutants, called “merovingian,” exhibit abnormal development of hair cells due to increased acidification of the extracellular space in which they develop.^14^ We sought to determine if the acidification of the otic placode was distinguishable in our Tg(Ubi:pHluorin2‐GPI) model system. We observed a distinct peak in proton concentrations at 24 hpf within the developing inner ear (Figure 3A). However, this peak disappeared by 48 hpf and was not observed at 72 hpf (Figures 3B,C and S5C). We observed two distinct proton populations at 24 hpf. The first population consists of a diffuse signal along the outer layer of the otic placode (Figure 3D,E). A second more intense population consists of a localized signal in the medial region of the otic placode (Figure 3D,F). Our findings provide evidence of a transient acidification event with distinct boundaries that may play an important role in the development of hair cells.^32^, ^33^, ^34^

### 2.4 The extracellular acidification of ionocytes

**FIGURE 4:**
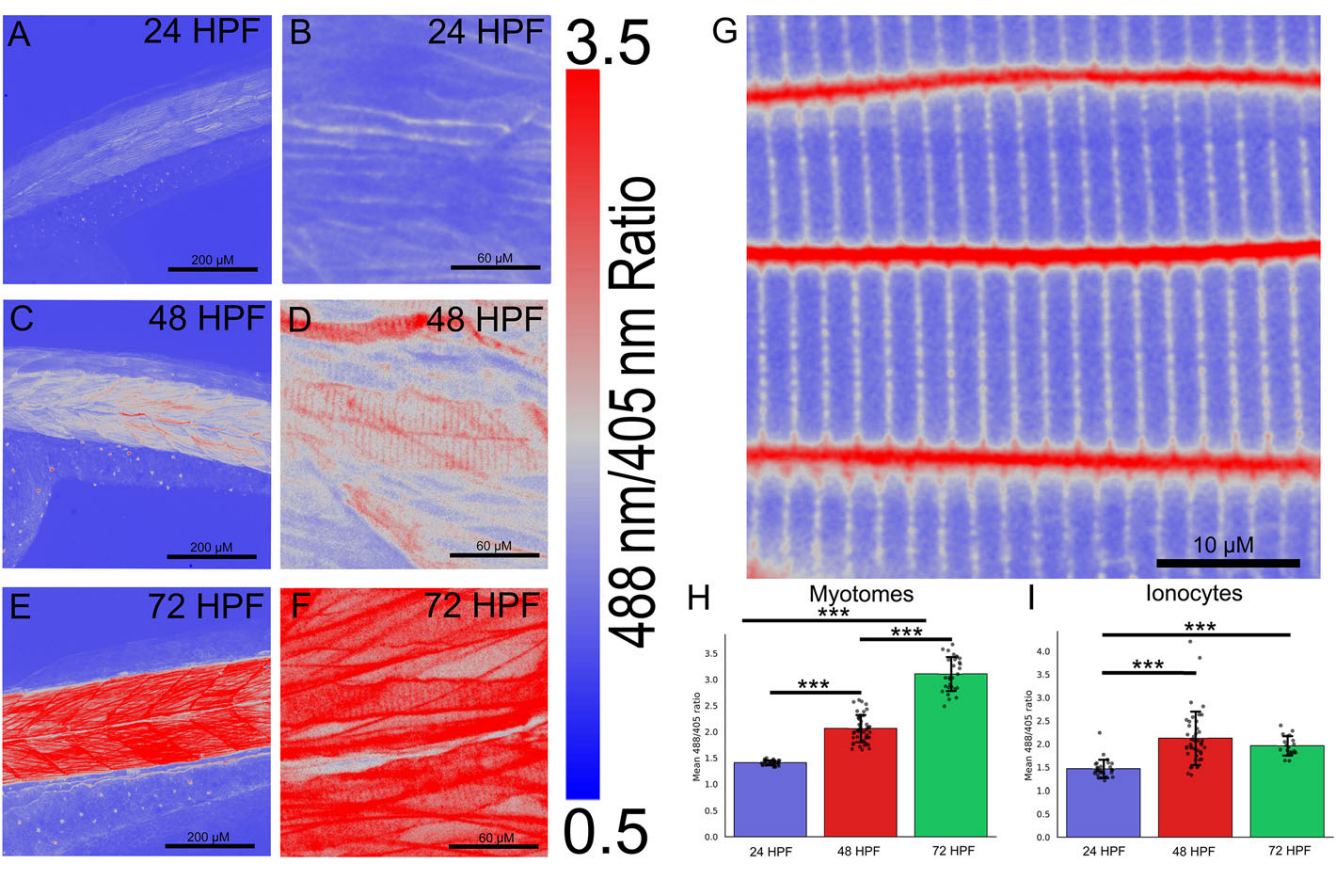
Acidification of the larval myotome. (A) and (B) At 24 hpf there is a little acidification around the myocytes in the trunk, with individual rounded cells primarily along the yolk. (C) By 48 hpf the level of acidification around the myocytes has increased significantly. (D) Closer examination reveals that the more acidified regions have more prominent T‐tubules. (C) and (E) Scattered cells along the yolk are still observed. (E) and (F) 72 hpf the myotome has become highly acidic with more prominently visible T‐tubules. (G) A single plane through the myotome of a 72 hpf embryo at higher resolution. This image shows that the bulk of myotome acidification is between the myotomes and not in the T‐tubules. (H) Quantification of the progressive increase in 488/405 ratios over time in the myotome. (I) Ionocytes on the yolk increase in 488/405 ratio from 24 to 48 hpf but not from 48 to 72 hpf. (A), (C), and (E) Punctate cells ventral to the muscles are ionocytes. (A–F) Representative images from *n* ≥ 3 biological repeats. (H) 5–8 regions of interest across 3–5 embryos for a minimum *n* ≥ 29 and (I) 5–8 ionocytes measured across 3–5 embryos for a minimum *n* ≥ 20 (A–F) 20× magnification. (B), (D), and (F) are magnified insets from (A), (C), and (E). (G) 63× magnification. ***p < .001.

Larval zebrafish rely on a special set of cells called ionocytes to maintain a healthy balance of ions and acids in their bodies. These ionocytes reside in the epidermis, the outer layer of their skin, acting as a crucial substitute for gills that have not yet fully developed. The key function of ionocytes is to regulate larval pH by controlling the movement of hydrogen ions (H^+^) and bicarbonate ions (HCO_3_^−^) across the skin. We easily identified the ionocytes in Tg(phlourin2‐GPI) due to their high activity, pumping acid out of the body by 24 hpf. (Figures 3B,C, 4A,C,E, and S6). The activity of these ionocytes ramped up until it reached a plateau at 48 hpf and activity remained constant at 72 hpf (Figure 4I). Our data suggests ionocytes functionality increases until it plateaus at 48 hpf.

### 2.5 The compartmentalized extracellular acidification of the myotome

Lactic acid accumulation is seen physiologically in developed muscles in response to exercise. We similarly saw proton burden increase during development. In Tg(Ubi:pHluorin2‐GPI) embryos, we observed a mild increase in extracellular acidification around myocytes at 24 hpf (Figure 4A,B). The acidification becomes more pronounced by 48 hpf, coinciding with the growth of T‐tubules within the more mature muscle cells (Figure 4A,B).^35^ We noted that at 48 hpf, the level relative of acidity varies between individual muscle cells (Figure 4C,D). By 72 hpf, myocytes exhibit a significant increase in extracellular acidification (Figure 4E,F). This pattern is not limited to the trunk muscles as facial and fin muscles also show a similar rise in extracellular acidification (Figure 3B,C). Notably the use of GPI anchor, which is known to localize fluorophores to T‐tubules allowed us to look at the fine structure in our model.^35^ Our high‐resolution observations revealed that T‐tubules themselves appear less acidic compared to the general area around the muscle cells (intermyocyte space) (Figures 4G and S7).^35^ This data suggests potential spatial compartmentalization of proton concentration within the muscle microenvironment regulated by T‐tubules.

### 2.6 Myotome proton burden is dependent on muscle activity

**FIGURE 5:**
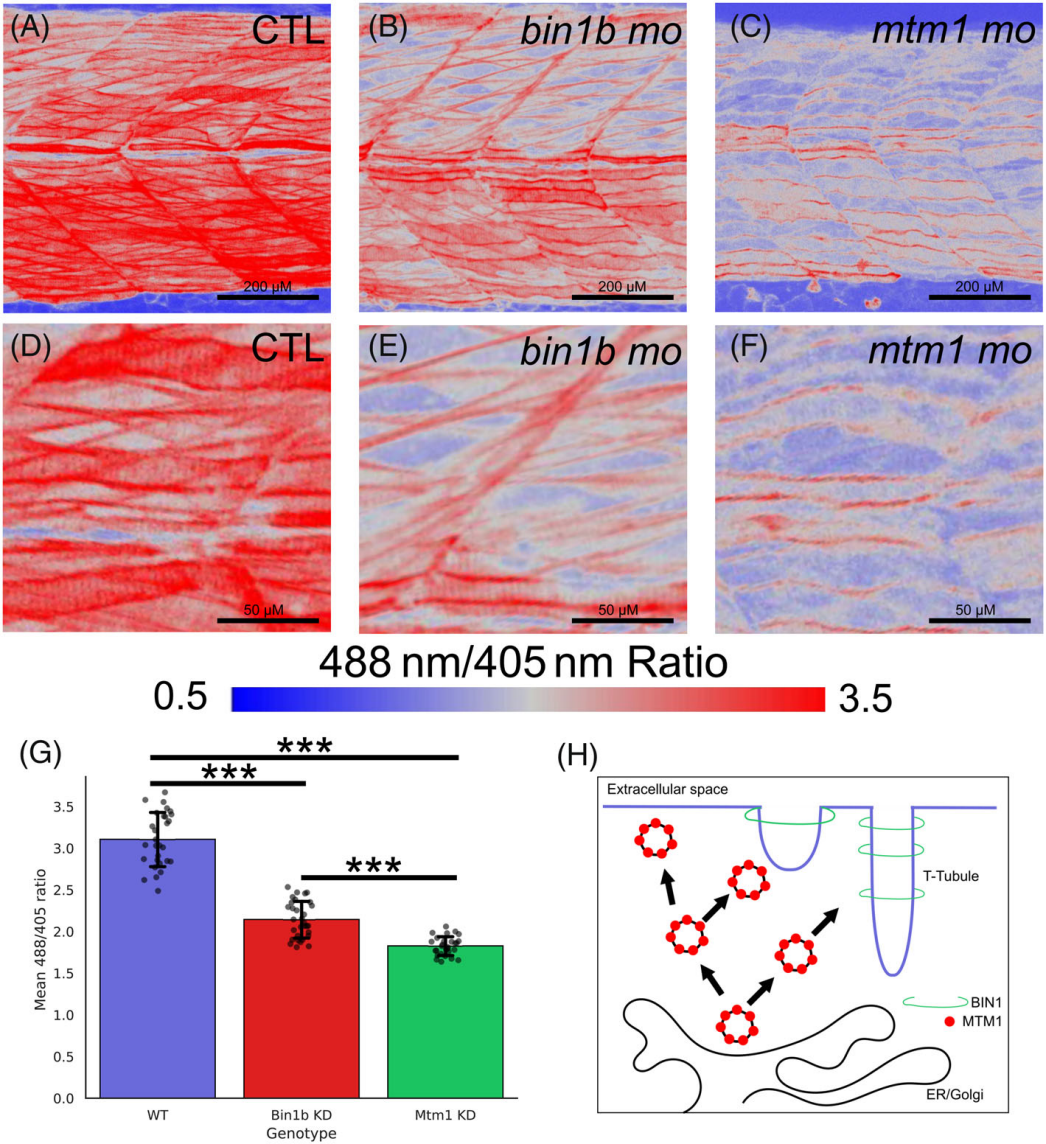
Acidification of the myotome is dependent on proper t‐tubule formation. (A–C) Knockdown of bin1b and mtm1 result in decreased acidification in the myotome. (D–F) Higher magnification images of the bin1b and mtm1 knockdowns demonstrate malformation and reduction in the number of T‐tubules. (G) Quantification of reduced 488/405 ratios across control and knockdowns. (H) Schematic for the role of bin1b and mtm1 in T‐tubule formation. Bin1 (Amphiphysin 2) acts as a scaffolding protein, initiating membrane invagination and stabilizing newly formed T‐tubules. MTM1 (Myotubularin 1) is crucial for Bin1‐induced tubulation. It maintains phosphoinositide homeostasis within the cell, creating a favorable environment for T‐tubule development. (A–F) Representative images from *n* ≥ 3 biological repeats. (G) 5–8 regions of interest across 3–5 embryos for a minimum *n* ≥ 29 (A–C) 20× magnification. (D–F) are magnified insets from (A–C); ***p < .001.

**FIGURE 6:**
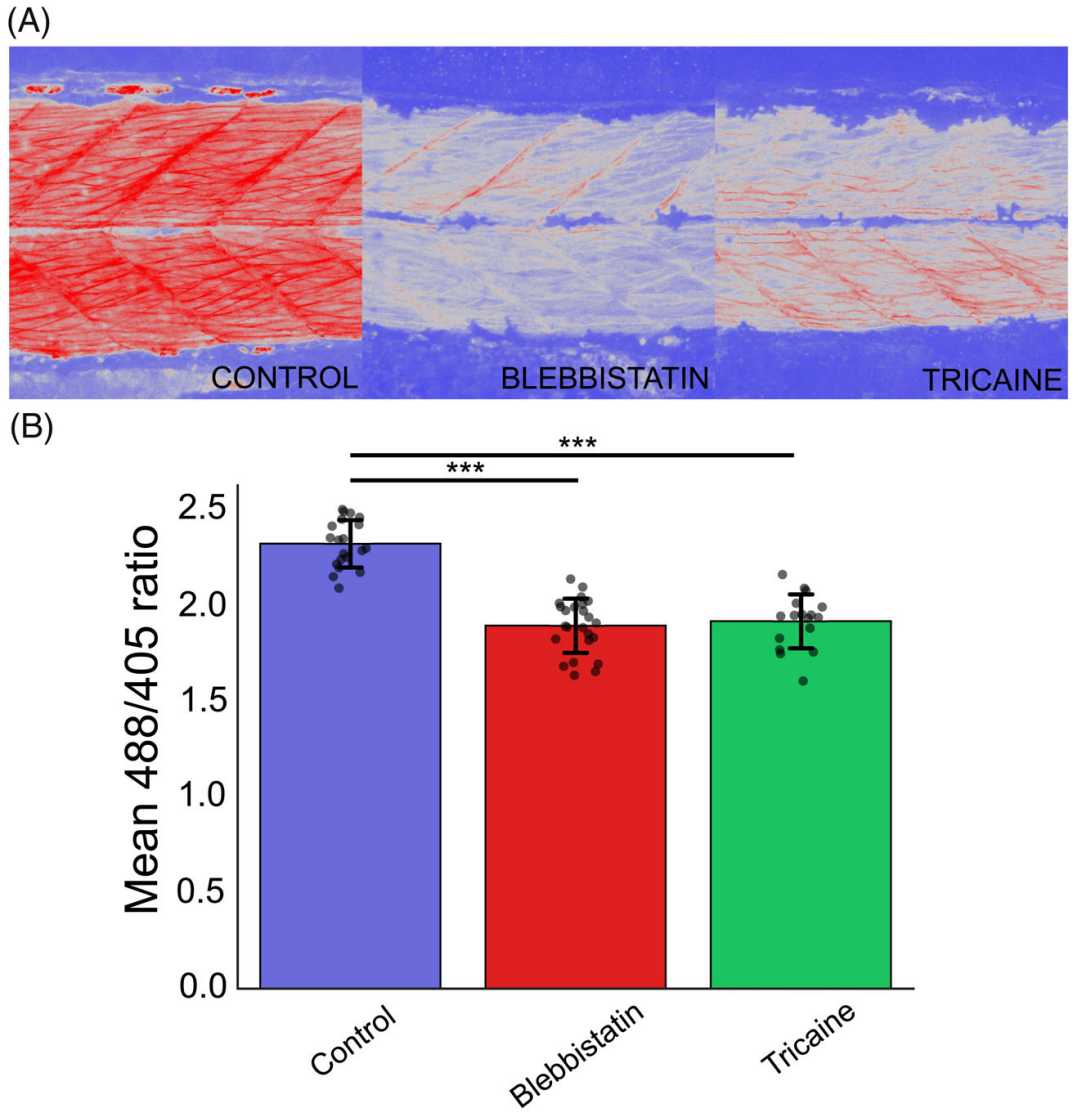
Pharmacological immobilization of zebrafish reduces acidification in the myotome. Twenty‐four hours postfertilization embryos were treated with blebbistatin or tricaine for 48 h. (A) Sagittal images of the distal tail in 72 hpf showed significant decreases in myotome acidification. (B) Quantification of 488/405 ratios in drug treatment groups. (A) Representative images from *n* ≥ 3 biological repeats; 20× magnification. (B) 5–8 regions of interest across 3–5 embryos for a minimum *n* ≥ 28; ***p < .001.

Given the significant increase in extracellular acidification with the emergence of T‐tubules we sought to test if the T‐tubules have a role in the acidification process. The loss of T‐tubule components like bin1 and mtm1 results in the human disease known as centronuclear myopathy, which manifests with symptoms of myalgia, motor symptoms, respiratory symptoms, and exercise intolerance.^36^ When bin1b and mtm1 were knocked down the resulting extracellular acidification of the embryonic myotome was significantly decreased (Figures 5A–G and S8). The decrease in acidification could be due to the loss of T‐tubules or, analogous to acidification due to exercise, due to the diminished motility of the fish (Figure 5D–F). To differentiate between these two possibilities, we immobilized the embryos using two distinct pharmacological methods with prolonged exposure to blebbistatin (a myosin inhibitor) or MS‐222 (a sodium channel blocker) and assessed their extracellular acidification. We found a significant decrease in extracellular acidification in both treatment groups (Figure 6A,B). This data suggests that the extra cellular acidification of the myotome was due at least in part to myocyte activity and not wholly dependent on the structure of the T‐tubule.

## 3 DISCUSSION

The role of extracellular protons in the context of signaling molecules remains relatively unexplored, especially during development. However, studies in other contexts, such as inflammation and cancer, highlight their importance in injury response and disease signaling. Our lab discovered OGM, a specific inhibitor of GPR68, a proton‐sensing G protein‐coupled receptor, in a phenotype‐based developmental screen on zebrafish. The resulting phenotype from treatment during gastrulation suggested that extracellular acid sensing plays a key role in the development of the notochord, craniofacial cartilage, and pigmentation.^16^ The importance of extracellular acidification in signaling is bolstered by findings in *C. elegans* gut function and mammalian inflammatory diseases.^1^, ^4^ Furthermore, work by others on V‐ATPase in craniofacial development and zebrafish notochord function suggests their potential involvement.^12^, ^13^, ^16^ Therefore, it is likely that extracellular protons and their detection play multiple spatially and temporally relevant roles in developing embryos.

Limitations of various tools have hampered the study of extracellular protons. Many studies use BCECF, a pH sensitive dye, which is prone to heterogeneous uptake and is easily photobleached, which limits its use to durations <60 minutes. By contrast, our use of a genetically‐encoded pH sensor will allow even distribution of the fluorophore and longer imaging with reduced photobleaching. This study introduces the Tg(ubi:pHluorin2‐GPI) transgenic line as a novel tool and approach to investigate extracellular proton dynamics during zebrafish development. This line expresses a ratiometric pH‐sensitive fluorescent sensor anchored specifically on the cell membrane to enable visualization of dynamic extracellular proton burden. Employing this tool, we observed spatiotemporal extracellular acidification patterns in diverse tissues including the notochord, otic placode, and skeletal muscle.

We observed regions of high proton burden emerge along the lamina of the notochord. Williams et al. discovered inhibiting the proton sensing GPCR, GPR68, resulted in an undulating or wavy notochord, suggesting a vital role for proton burden in the lamina during notochord formation.^16^ The protons generated at these foci are sensed by GPR68 to regulate the formation of the notochord.

The otic placode exemplifies the complexity of the regional extracellular acidification. Our data shows a clear dynamic change in proton burden throughout development. The peak relative acidification was observed at 24 hpf which then decreased to a stable level from 48 to 72 hpf. Furthermore, two distinct regions of extracellular acidification were evident. The first region was more broad, uniform, and lateral to the head. The second was a discrete boundary medial to the otic placode. This suggests a temporal and spatial requirement for proton burden in the developing zebrafish ear.

The extracellular acidification of muscles exhibited an increase and intensification coinciding with the emergence of T‐tubules. The observed acidic domains were sensitive to genetic disruption of bin1b and mtm1 which are critical to t‐tubule formation and homeostasis. Furthermore, the myocyte acidification was sensitive to disruption with both blebbistatin, a myosin inhibitor, and tricaine, a sodium channel blocker, suggesting that the increased protons came from muscle activity and not the T‐tubules themselves. As we observed that the relative acidification is significantly higher in the intermyocyte space compared to the t‐tubule; we hypothesize that the T‐tubule maintains a specific ionic balance by excluding elevated proton levels from muscle activity. The physiological importance of extracellular pH in relation to T‐tubules lies in its role in modulating excitation‐contraction coupling. The activity of voltage‐gated calcium channels located on the T‐tubules is highly dependent on pH. These channels are responsible for triggering calcium release from the sarcoplasmic reticulum, a critical step in muscle contraction.^37^, ^38^, ^39^, ^40^, ^41^, ^42^, ^43^, ^44^ Taken together our data suggests that the T‐tubule helps maintain proper ionic balance (inclusive of H^+^), which is critical for proper channel activity, and calcium release, and ultimately leads to robust contractile force generation.

We have developed a new tool, Tg(ubi:pHluorin2‐GPI), and observed changes in extracellular proton localization throughout early zebrafish development. While these findings unveil novel roles for extracellular protons in zebrafish development several questions remain unanswered. Future studies utilizing subcellular pH sensors and electrophysiological techniques could provide deeper insights into specific proton burden dynamics within T‐tubules, the otic vesical, notochord, and other microenvironments. Elucidating how cells generate, respond to, and utilize these pH changes could significantly enhance our understanding of muscle function and various diseases. Ultimately, this knowledge could pave the way for improved therapies for muscle, spinal, and auricular diseases as well as strategies for optimizing exercise performance.

## 4 EXPERIMENTAL PROCEDURES

### 4.1 Zebrafish

Zebrafish embryos were obtained by natural spawning and maintained on a 14‐h light/10‐h dark cycle at 28°C according to standard procedures. Embryos were raised in E3 media supplemented with 0.25 mg/mL methylene blue and staged based on either their somite count or hours postfertilization (hpf). If required, pigmentation was inhibited by adding 0.003% (w/v) 1‐phenyl‐2‐thiourea (PTU) to the E3 media at 24 hpf. Animal protocols were approved by the University of Maryland Baltimore and Michigan State University.

### 4.2 Generation of ubi:pHluorin2‐GPI allele

The Tol2(ubi:pHluorin2‐GPI) construct was synthesized by VectorBuilder (VB200601‐1084rcb; Supplemental File 1). The promoter sequence for zebrafish Ubiquitin was used, followed by pHluorin2, a GPI localization sequence.^14^, ^19^, ^45^, ^46^ The construct was flanked by Tol2 element sequence sites to allow transgenesis. Transposase mRNA was in vitro transcribed using the mMessage mMachine T7 Ultra Transcription Kit (Invitrogen) from AscI linearized Tol2 plasmid (VectorBuilder: VB220830‐1237vmx; Supplemental File 1). Purified plasmid DNA was co‐injected, at a concentration of 20 ng/μL, with 80 pg. of Tol2 transposase mRNA into one‐cell zebrafish embryos. Resulting carriers were outcrossed and a single line was then bred to homozygosity prior to experimentation.

### 4.3 Morpholino oligonucleotide injection

In this study, we used the previously characterized antisense morpholinos against *mtm1* oligonucleotide (MO1‐mtm1: 5′‐AGACCCTCGTCGAAAAGTCATAACG‐3′) and *bin1b* oligonucleotide (MO1‐binb: 5′‐TGACTCCTTTCCCAACCTCTGCCAT‐3′). Alternatively, embryos were injected with the industry standard non‐targeting sequence (5′‐CCTCTTACCTCAGTTACAATTTATA‐3′) (Gene Tools, LLC).^47^, ^48^ Briefly, the non‐targeting sequence targets a human beta‐globin intron mutation that causes beta‐thalassemia and does not target any known sequence or cause any known phenotype in the zebrafish. Embryos were injected in the yolk with 6 ng MO in 3 nL at the single‐cell stage. Embryos were then grown as described above.

### 4.4 Keratocyte explants

Adult scales were collected as described in Rapanan et al.^33^ Scales were placed in FluoroBrite DMEM with 10% FBS and penicillin–streptomycin and imaged on an EVOS M5000 microscope (Thermofisher).

### 4.5 Heterozygous comparisons

Homozygous Tg(ubi:pHluorin2‐GPI) were outcrossed to wild type AB fish. Three days postfertilization samples were anesthetized with tricaine methanesulfonate for 1 h and imaged.

### 4.6 4% PFA fixation

Live 3 dpf samples were anesthetized with tricaine for 30 min and imaged. Samples were then fixed in 4% Paraformaldehyde in PBS (pH 7.4) for 2 h at room temperature with gentle rocking. Fixed samples were then reimaged.

### 4.7 Nigericin treatments

Three days postfertilization embryos were transferred to a 96 well plate with fresh E3 media containing tricaine and allowed to rest for 3 h at 28.5°C before imaging. Nigericin was added to the wells for a final concentration of 100 μM. Samples were then incubated for 30 min at 28.5°C, briefly washed with fresh E3, and reimaging.

### 4.8 Prolonged blebbistatin and tricaine treatment

At 24 hpf embryos were manually dechorionated and separated into treatment groups of DMSO (0.1%), blebbistatin (10 μM), or tricaine (0.016%) in E3 media containing for 48 h before imaging. At 72 hpf fish were prepared for imaging as described below.

### 4.9 pH media treatments

Three days postfertilization embryos were transferred to a 96 well plate with fresh E3 media containing tricaine at pH 4 or 8. Samples were incubated for 3 h at 28.5°C before imaging.

### 4.10 SBFI sodium staining

H^+^‐ATPase‐rich (HR) ionocytes were stained at 3 dpf with SBFI AM cell permeant dye (Invitrogen).^33^, ^49^ Embryos were incubated in 10 μM SBFI in E3 media for 1 h at 28.5°C. Larvae were washed with fresh E3, anesthetized in tricaine, and embedded in 2% low melting point agarose in E3 media for imaging.

### 4.11 Image acquisition

Embryos were anesthetized with 0.016% tricaine in E3 medium, and then mounted laterally in low melt agarose. All mounted fish were imaged within 3 h of mounting. Whole mount image Figure 1C was acquired on a LionheartFX (BioTek). Figures 1E and 2, 3, 4, 5 were imaged with a Yokogawa CSU‐W1 spinning disk confocal microscope from Nikon. Spinning disk images were captured as Z‐stacks at 20× (0.75 NA) or 63× (1.49 NA) magnification at consistent exposure time and excitation to allow for comparisons between samples. The average depth for image capture of z‐stacks was 74 μm. Figures [Link], [Link], and S8 were captured on an Agilent BioTek Cytation C10. Figure S1 was acquired using an EVOS M5000 microscope (Thermofisher). Figure S6 was imaged with a Nikon Eclipse Ti2‐E.

### 4.12 Image analysis

All image analysis was done in Image J or the Gen5 Software from BioTek. For Figures 1E and 2, 3, 4, 5, a 3 × 3 pixel averaging smoothing was applied to all images. Using Image J, 488ex/525em images were then divided by 405ex/525em images to obtain a ratiometric images. Pseudo‐coloring for ratios was done with 0.5 minimum and 3.5 maximum for all images. For t‐tubules measurements Inter myocyte space was defined as the long edge between two myocytes along the axis above the yolk extension, t‐tubules were the perpendicular protrusion into myocytes, inter t‐tubule space was the measure within the cell between the t‐tubules.

### 4.13 Statistical analysis

RStudio or Python, seaborn package was used to draw graphs, error bars represent the standard deviation. R 4.3.1 was used to calculate all statistics. Comparisons were done using an unpaired, two‐tailed Student's *t*‐test or one‐way ANOVA with Tukey–Kramer post hoc analysis.

## CONFLICT OF INTEREST STATEMENT

The authors declare no conflicts of interest.

## Supporting information


**Figure S1.** Keratocyte explants from adult zebrafish express pHlourin2‐GPI. Keratocyte from adult zebrafish express pHlourin2‐GPI. Representative images of a 1 year old fish from *n* ≥ 3 biological repeats with *n* ≥ 20 fish. 20× magnification.

**Figure S2.** Heterozygotes express half as much pHluorin2‐GPI as homozygotes. (A) Representative 405 nm and 488 nm images from Tg(ubi:pHluorin2‐GPI) homozygotes, AB control homozygotes, and Tg(ubi:pHluorin2‐GPI) × AB heterozygotes. 10× magnification of 3 dpf embryos. (A) Quantification of 405 nm expression of pHluorin2‐GPI. Total intensity of whole embryos normalized to Tg(ubi:pHluorin2‐GPI) homozygotes. Control embryos represent yolk and pigment cells autofluorescence. (B) *n* ≥ 33 fish aggregated from *n* = 3 biological repeats. ***p < 0.001.

**Figure S3.** pHlourin responds to environmental stimuli. (A) Tg(ubi:pHluorin2‐GPI) samples pre‐ and post‐treatment with the potassium ionophore Nigericin. 20× magnification. (B) Quantification of pH dysregulation caused by Nigericin treatment. (Left) pH independent fluorescence was unchanged but (Right) average embryo pH increased significantly. (C) Embryos soaked at pH 4 show higher global acidity than embryos at pH 8. (Left) 488 nm fluorescence increases in the acidic condition while (Middle) 405 nm fluorescence decreases. (Right) global fluorescence across the embryo is significantly increased in the acidic environment. (B) Representative data from *n* = 4 biological repeats with *n* = 45 embryos; normalized to pre‐treatment. (C) Data aggregated from *n* ≥ 4 biological repeats with *n* ≥ 62 embryos; normalized to pH 8. ***p < .001.

**Figure S4.** 4% PFA fixation increases background. Representative images of Tg(ubi:pHluorin2‐GPI) embryos at 3 dpf pre‐ and post‐fixation. 2× magnification. *n* ≥ 3 biological replicates with *n* ≥ 10 embryos.

**Figure S5.** Quantification of notochord lamina and otic placode. Acidification of the inter‐vacuole lamina along the (A) anterior–posterior axis and (B) dorsal‐ventral axis of the notochord increases from 24 to 72 hpf. (C) Acidification of the otic placode decreases from 24 to 72 hpf. (A) and (B) aggregate measurements of 5 different inter‐vacuole lamina from *n* = 6 embryos in *n* = 3 biological repeats. (C) *n* = 5 embryos from *n* = 3 biological replicates. (A–C) normalized to 24 hpf; * *p* < .05, ***p < .001.

**Figure S6.** pHlourin2‐GPI expression in ionocytes. (A) SBFI labeling of sodium rich H^+^‐ATPase‐rich (HR) ionocytes around the yolk sac coincides with high 488 nm signaling of pHlourin2‐GPI. Arrows indicate an ionocyte. (B) Magnified inset from Figure 3B.

**Figure S7.** Intermyocyte space is more proton‐rich than the T‐tubules. Quantification of 488/405 ratios at high resolution from Figure 4G shows that the intermyocyte space has a higher proton burden than the T‐tubules. Data aggregated from *n* = 3 embryos with each with 3–4 regions of interest for a minimum *n* ≥ 10; normalized to the inter‐t‐tubule space. ***p < .001.

**Figure S8.** Bin1b and Mtm1 decrease extracellular acidification of the embryonic myotome. (A) Whole animal photos of control MO, Bin1b MO, and Mtm1 MO in comparison to wild type controls. Knockdown of Bin1b and Mtm1 decrease expression of pHlourin2 at 72 hpf. (B) Quantification of all gross morphological changes in MO injected and WT groups. No repeatable gross malformations were observed, and no significant differences were observed between groups. (B) Averages of *n* = 3 biological replicates and a total of *n* ≥ 101 embryos from all groups combined.

Supplementary File 1.

## Data Availability

The data sets and zebrafish lines used and/or analyzed during the current study are available from the corresponding author upon reasonable request.

## References

[1] Beg AA , Ernstrom GG , Nix P , Davis MW , Jorgensen EM . Protons act as a transmitter for muscle contraction in *C. elegans* . Cell. 2008;132:149‐160. doi:10.1016/j.cell.2007.10.058 18191228 PMC2258244

[2] Wyder L , Suply T , Ricoux B , et al. Reduced pathological angiogenesis and tumor growth in mice lacking GPR4, a proton sensing receptor. Angiogenesis. 2011;14:533‐544. doi:10.1007/s10456-011-9238-9 22045552

[3] Wang Y , de Vallière C , Imenez Silva PH , et al. The proton‐activated receptor GPR4 modulates intestinal inflammation. J Crohns Colitis. 2018;12:355‐369. 29136128 10.1093/ecco-jcc/jjx147

[4] Castellone RD , Leffler NR , Dong L , Yang LV . Inhibition of tumor cell migration and metastasis by the proton‐sensing GPR4 receptor. Cancer Lett. 2011;312:197‐208. doi:10.1016/j.canlet.2011.08.013 21917373

[5] Wirasinha RC , Vijayan D , Smith NJ , et al. GPR65 inhibits experimental autoimmune encephalomyelitis through CD4+ T cell independent mechanisms that include effects on iNKT cells. Immunol Cell Biol. 2018;96:128‐136. doi:10.1111/imcb.1031 29363187

[6] Wang T , He M , Zha X‐M . Time‐dependent progression of hemorrhagic transformation after transient ischemia and its association with GPR68‐dependent protection. Brain Hemorrhages. 2020;1:185‐191. 33575546 10.1016/j.hest.2020.10.001 PMC7872135

[7] Xu Y , Lin MT , Zha XM . GPR68 deletion impairs hippocampal long‐term potentiation and passive avoidance behavior. Mol Brain. 2020;13:132. 32993733 10.1186/s13041-020-00672-8 PMC7526169

[8] Yan L , Singh LS , Zhang L , Xu Y . Role of OGR1 in myeloid‐derived cells in prostate cancer. Oncogene. 2012;33:157‐164. doi:10.1038/onc.2012.566 23222714

[9] De Vallière C , Bäbler K , Busenhart P , et al. A novel OGR1 (GPR68) inhibitor attenuates inflammation in murine models of colitis. Inflamm Intest Dis. 2021;6:140‐153. doi:10.1159/000517474 34722644 PMC8527911

[10] Zhang W , Han Y , Li W , et al. Clinical data analysis reveals the role of OGR1 (GPR68) in head and neck squamous cancer. Anim Models Exp Med. 2020;3:55‐61. 10.1002/ame2.12105 PMC7167242 32318660

[11] Guissart C , Li X , Leheup B , et al. Mutation of SLC9A1, encoding the major Na^+^/H^+^ exchanger, causes ataxia–deafness Lichtenstein–Knorr syndrome. Hum Mol Genet. 2015;24:463‐470. doi:10.1093/HMG/DDU461 25205112

[12] Vandenberg LN , Morrie RD , Adams DS . V‐ATPase‐dependent ectodermal voltage and Ph regionalization are required for craniofacial morphogenesis. Dev Dyn. 2011;240:1889‐1904. doi:10.1002/dvdy.22685 21761475 PMC10277013

[13] Pottie L , van Gool W , Vanhooydonck M , et al. Loss of zebrafish atp6v1e1b, encoding a subunit of vacuolar ATPase, recapitulates human ARCL type 2C syndrome and identifies multiple pathobiological signatures. PLoS Genet. 2021;17:e1009603. doi:10.1371/JOURNAL.PGEN.1009603 34143769 PMC8244898

[14] Stawicki TM , Owens KN , Linbo T , Reinhart KE , Rubel EW , Raible DW . The zebrafish merovingian mutant reveals a role for pH regulation in hair cell toxicity and function. Dis Models Mech. 2014;7:847‐856. doi:10.1242/dmm.016576 PMC4073274 24973752

[15] Hanaoka R , Ohmori Y , Uyemura K , et al. Zebrafish gcmb is required for pharyngeal cartilage formation. Mech Dev. 2004;121:1235‐1247. doi:10.1016/J.MOD.2004.05.011 15327784

[16] Williams CH , Neitzel LR , Cornell J , et al. GPR68‐ATF4 signaling is a novel prosurvival pathway in glioblastoma activated by acidic extracellular microenvironment. Exp Hematol Oncol. 2024;13:13. doi:10.1186/S40164-023-00468-1 38291540 PMC10829393

[17] Decoursey TE . Voltage‐gated proton channels and other proton transfer pathways. Am Physiol Soc. 2003;83:475‐579. doi:10.1152/physrev.00028.2002 12663866

[18] DeCoursey TE . Interactions between NADPH oxidase and voltage‐gated proton channels: why electron transport depends on proton transport. FEBS Lett. 2003;555:57‐61. doi:10.1016/S0014-5793(03)01103-7 14630319

[19] Mahon MJ . pHluorin2: an enhanced, ratiometric, pH‐sensitive green florescent protein. Adv Biosci Biotechnol. 2011;02:132‐137. doi:10.4236/abb.2011.23021 PMC3152828 21841969

[20] Kawakami K . Transposon tools and methods in zebrafish. Dev Dyn. 2005;234:244‐254. doi:10.1002/dvdy.20516 16110506

[21] Suster ML , Kikuta H , Urasaki A , Asakawa K , Kawakami K . Transgenesis in zebrafish with the tol2 transposon system. Methods Mol Biol. 2009;561:41‐63. doi:10.1007/978-1-60327-019-9_3 19504063

[22] Yang F , Moss LG , Phillips GN . The molecular structure of green fluorescent protein. Nat Biotechnol. 1996;14:1246‐1251. doi:10.1038/nbt1096-1246 9631087

[23] Sevcsik E , Brameshuber M , Fölser M , Weghuber J , Honigmann A , Schütz GJ . GPI‐anchored proteins do not reside in ordered domains in the live cell plasma membrane. Nat Commun. 2015;6:1‐10. doi:10.1038/ncomms7969 PMC4430820 25897971

[24] Navis A , Bagnat M . Developing pressures: fluid forces driving morphogenesis. Curr Opin Genet Dev. 2015;32:24‐30. doi:10.1016/J.GDE.2015.01.010 25698116 PMC4470832

[25] Ellis K , Bagwell J , Bagnat M . Notochord vacuoles are lysosome‐related organelles that function in axis and spine morphogenesis. J Cell Biol. 2013;200:667‐679. doi:10.1083/JCB.201212095 23460678 PMC3587825

[26] Ellis K , Hoffman BD , Bagnat M . The vacuole within: how cellular organization dictates notochord function. Bioarchitecture. 2013;3:64‐68. doi:10.4161/BIOA.25503 23887209 PMC3782541

[27] Lorente‐Cánovas B , Ingham N , Norgett EE , Golder ZJ , Frankl FEK , Steel KP . Mice deficient in H^+^‐ATPase a4 subunit have severe hearing impairment associated with enlarged endolymphatic compartments within the inner ear. Dis Model Mech. 2013;6:434‐442. doi:10.1242/DMM.010645 23065636 PMC3597025

[28] Su Y , Zhou A , Al‐Lamki RS , Karet FE . The a‐subunit of the V‐type H^+^‐ATPase interacts with phosphofructokinase‐1 in humans. J Biol Chem. 2003;278:20013‐20018. doi:10.1074/jbc.M210077200 12649290

[29] Su Y , Blake‐Palmer KG , Sorrell S , et al. Human H^+^ ATPase a4 subunit mutations causing renal tubular acidosis reveal a role for interaction with phosphofructokinase‐1. Am J Physiol Renal Physiol. 2008;295:F950‐F958. doi:10.1152/ajprenal.90258.2008 18632794 PMC2576143

[30] Forgac M . Vacuolar ATPases: rotary proton pumps in physiology and pathophysiology. Nat Rev Mol Cell Biol. 2007;8:917‐929. doi:10.1038/nrm2272 17912264

[31] Pamarthy S , Kulshrestha A , Katara GK , Beaman KD . The curious case of vacuolar ATPase: regulation of signaling pathways. Mol Cancer. 2018;17:41. doi:10.1186/s12943-018-0811-3 29448933 PMC5815226

[32] Chang WJ , Hwang PP . Development of zebrafish epidermis. Birth Defects Res C Embryo Today. 2011;93:205‐214. doi:10.1002/bdrc.20215 21932430

[33] Hwang PP , Chou MY . Zebrafish as an animal model to study ion homeostasis. Pflugers Arch. 2013;465:1233‐1247. doi:10.1007/s00424-013-1269-1 23568368 PMC3745619

[34] Lewis L , Kwong RWM . Zebrafish as a model system for investigating the compensatory regulation of ionic balance during metabolic acidosis. Int J Mol Sci. 2018;19:1087. doi:10.3390/ijms19041087 29621145 PMC5979485

[35] Hall TE , Martel N , Ariotti N , et al. In vivo cell biological screening identifies an endocytic capture mechanism for T‐tubule formation. Nat Commun. 2020;11:3711. doi:10.1038/S41467-020-17486-W 32709891 PMC7381618

[36] Reumers SFI , Erasmus CE , Bouman K , et al. Clinical, genetic, and histological features of centronuclear myopathy in the Netherlands. Clin Genet. 2021;100:692‐702. doi:10.1111/CGE.14054 34463354 PMC9292987

[37] Stengl M , Carmeliet E , Mubagwa K , Flameng W . Modulation of transient outward current by extracellular protons and Cd^2+^ in rat and human ventricular myocytes. J Physiol. 1998;511:827‐836. doi:10.1111/j.1469-7793.1998.827bg.x 9714863 PMC2231156

[38] Saegusa N , Moorhouse E , Vaughan‐Jones RD , Spitzer KW . Influence of ph on Ca^2+^ current and its control of electrical and Ca^2+^ signaling in ventricular myocytes. J Gen Physiol. 2011;138:537‐559. doi:10.1085/jgp.201110658 22042988 PMC3206307

[39] Garciarena CD , Ma YL , Swietach P , Huc L , Vaughan‐Jones RD . Sarcolemmal localisation of Na^+^/H^+^ exchange and Na^+^‐HCO_3_ ^−^ co‐transport influences the spatial regulation of intracellular pH in rat ventricular myocytes. J Physiol. 2013;591:2287‐2306. doi:10.1113/jphysiol.2012.249664 23420656 PMC3650695

[40] Tombaugh GC , Somjen GG . Effects of extracellular pH on voltage‐gated Na^+^, K^+^ and Ca^2+^ currents in isolated rat CA1 neurons. J Physiol. 1996;493:719‐732. doi:10.1113/jphysiol.1996.sp021417 8799894 PMC1159020

[41] Mačianskiene R , Almanaityte M , Jekabsone A , Mubagwa K . Modulation of human cardiac trpm7 current by extracellular acidic ph depends upon extracellular concentrations of divalent cations. PLoS One. 2017;12:e0170923. doi:10.1371/journal.pone.0170923 28129376 PMC5271359

[42] Yamamoto S , Ehara T . Acidic extracellular pH‐activated outwardly rectifying chloride current in mammalian cardiac myocytes. Am J Physiol Heart Circ Physiol. 2006;290:H1905‐H1914. doi:10.1152/ajpheart.00965.2005 16339831

[43] Hu YL , Mi X , Huang C , et al. Multiple H^+^ sensors mediate the extracellular acidification‐induced [Ca^2+^]_ *i* _ elevation in cultured rat ventricular cardiomyocytes. Sci Rep. 2017;7:44951. doi:10.1038/srep44951 28332558 PMC5362981

[44] Wu C , Fry CH . The effects of extracellular and intracellular pH on intracellular Ca^2+^ regulation in Guinea‐pig detrusor smooth muscle. J Physiol. 1998;508:131‐143. doi:10.1111/j.1469-7793.1998.131br.x 9490828 PMC2230873

[45] Caras IW , Weddell GN , Davitz MA , Nussenzweig V , Martin DW . Signal for attachment of a phospholipid membrane anchor in decay accelerating factor. Science. 1987;1979(238):1280‐1283. doi:10.1126/SCIENCE.2446389 2446389

[46] Mosimann C , Kaufman CK , Li P , Pugach EK , Tamplin OJ , Zon LI . Ubiquitous transgene expression and Cre‐based recombination driven by the ubiquitin promoter in zebrafish. Development. 2011;138:169‐177. doi:10.1242/DEV.059345 21138979 PMC2998170

[47] Hong TT , Smyth JW , Chu KY , et al. BIN1 is reduced and Cav1.2 trafficking is impaired in human failing cardiomyocytes. Heart Rhythm. 2012;9:812‐820. doi:10.1016/j.hrthm.2011.11.055 22138472 PMC3306544

[48] Dowling JJ , Vreede AP , Low SE , et al. Loss of myotubularin function results in T‐tubule disorganization in zebrafish and human myotubular myopathy. PLoS Genet. 2009;5:e1000372. doi:10.1371/JOURNAL.PGEN.1000372 19197364 PMC2631153

[49] Esaki M , Hoshijima K , Kobayashi S , Fukuda H , Kawakami K , Hirose S . Visualization in zebrafish larvae of Na^+^ uptake in mitochondria‐rich cells whose differentiation is dependent on foxi3a. Am J Physiol Regul Integr Comp Physiol. 2007;292:470‐480. doi:10.1152/AJPREGU.00200.2006/SUPPL_FILE/ESAKI_R-00200-2006-R2_TABLE_S1.PDF 16946087

