# Supplementary figures and images for: A novel transgenic reporter of extracellular acidification in zebrafish elucidates skeletal muscle T‐tubule pH regulation
Source: Dev Dyn. 2025 Jan 22;254(9):1068–79. doi: 10.1002/dvdy.770 (PMC12238821; doi:10.1002/dvdy.770)

Control

Tg(ubi:pHluorin2-GPI)

Bright Field

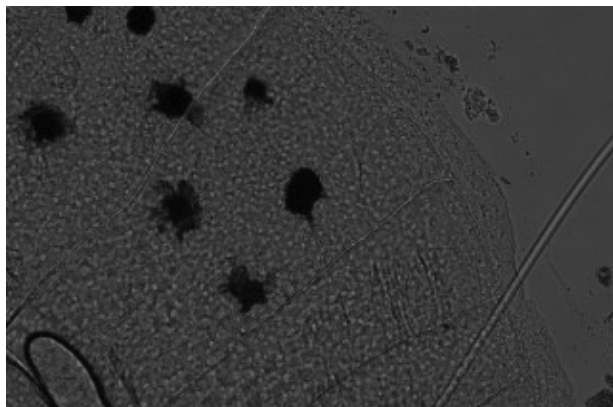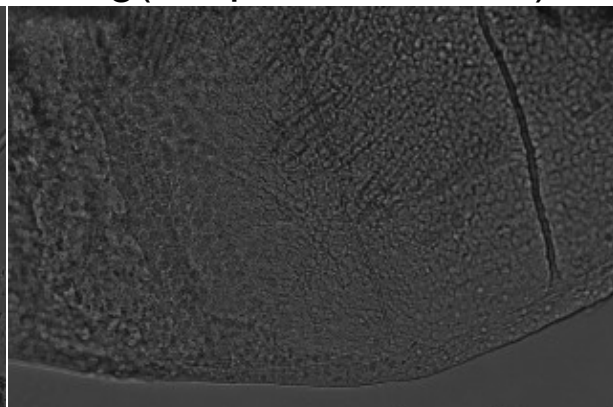

488 nm

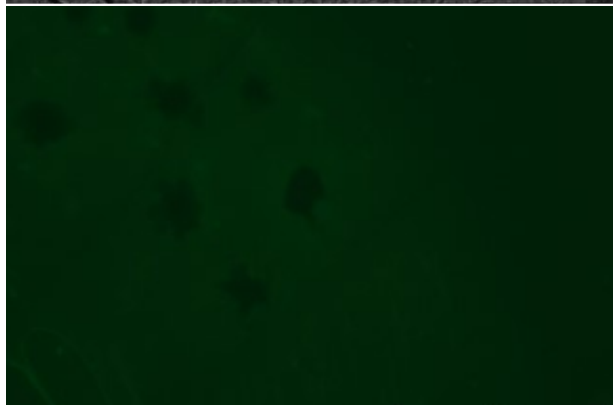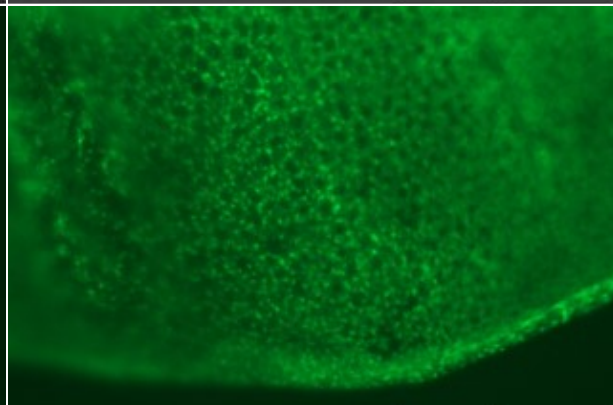

Supplement: Supplementary file 1 — Figure S1. Keratocyte explants from adult zebrafish express pHlourin2‐GPI. Keratocyte from adult zebrafish express pHlourin2‐GPI. Representative images of a 1 year old fish from n ≥ 3 biological repeats with n ≥ 20 fish. 20× magnification. [file DVDY-254-1068-s001.pdf]

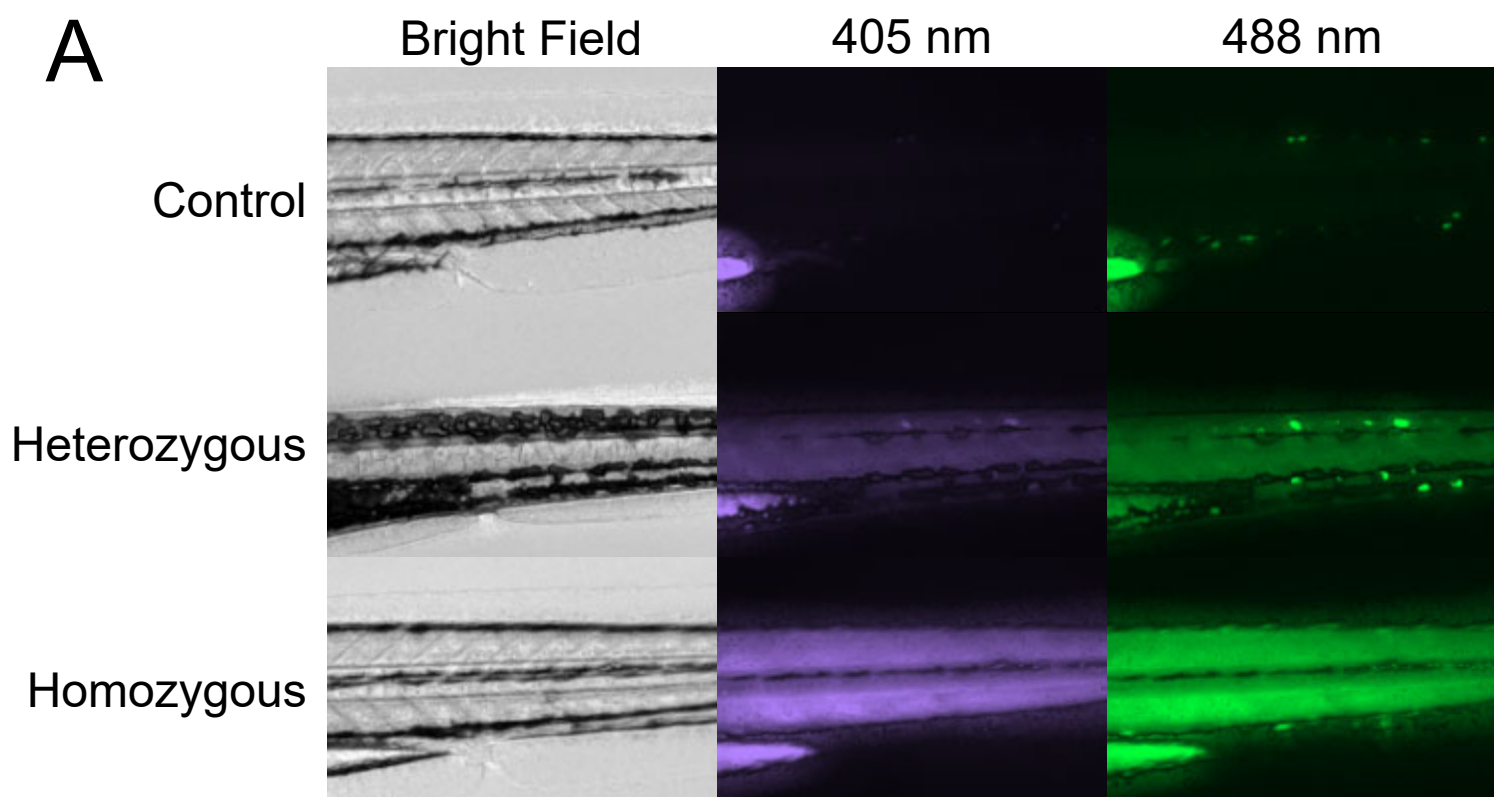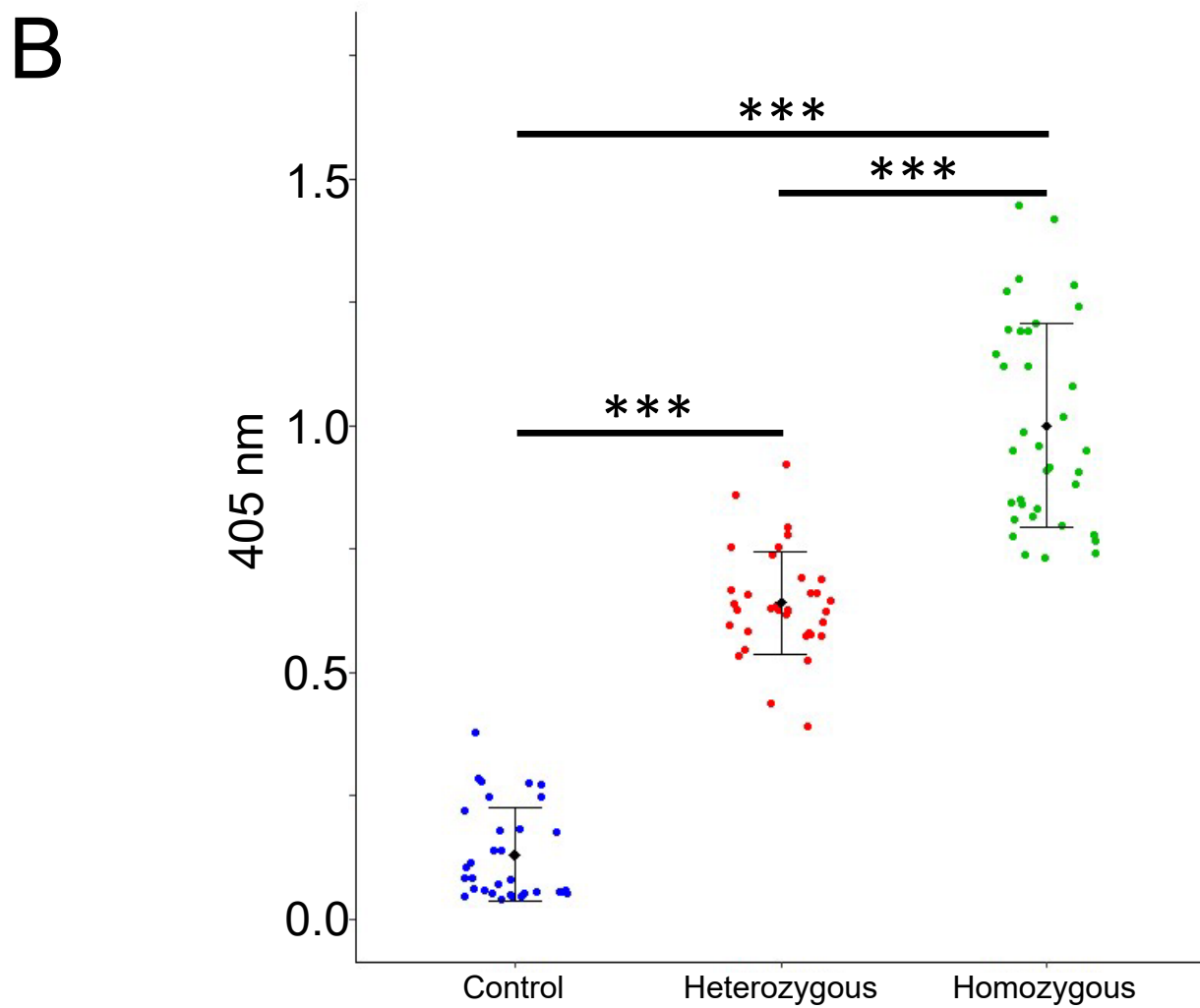

Supplement: Supplementary file 2 — Figure S2. Heterozygotes express half as much pHluorin2‐GPI as homozygotes. (A) Representative 405 nm and 488 nm images from Tg(ubi:pHluorin2‐GPI) homozygotes, AB control homozygotes, and Tg(ubi:pHluorin2‐GPI) × AB heterozygotes. 10× magnification of 3 dpf embryos. (A) Quantification of 405 nm expression of pHluorin2‐GPI. Total intensity of whole embryos normalized to Tg(ubi:pHluorin2‐GPI) homozygotes. Control embryos represent yolk and pigment cells autofluorescence. (B) n ≥ 33 fish aggregated from n = 3 biological repeats. ***p < 0.001. [file DVDY-254-1068-s007.pdf]

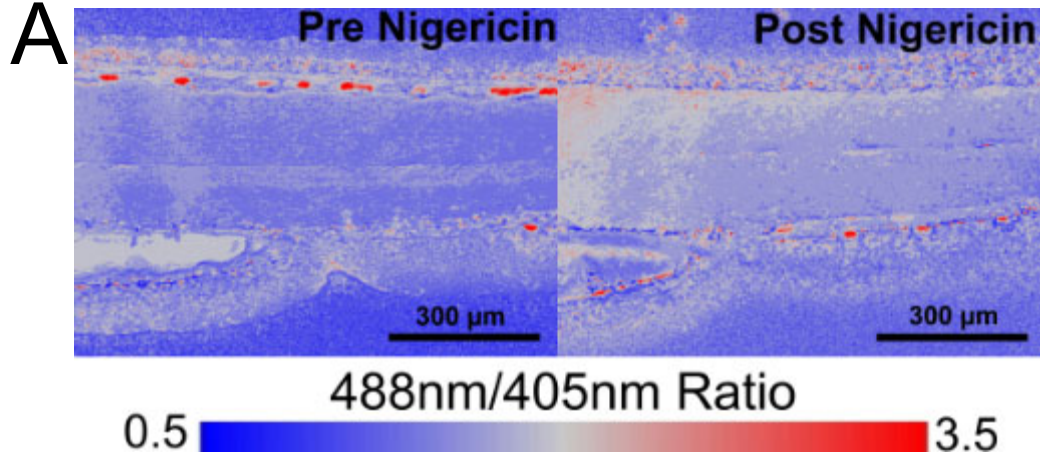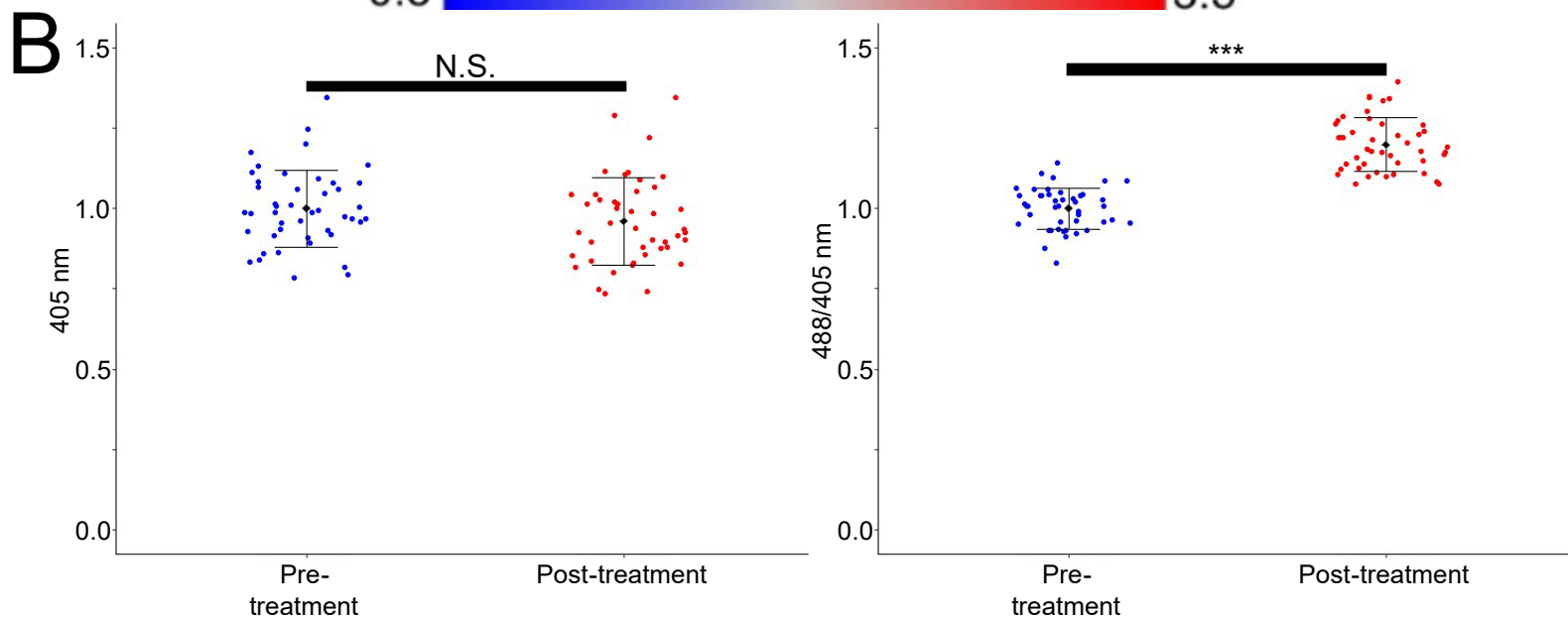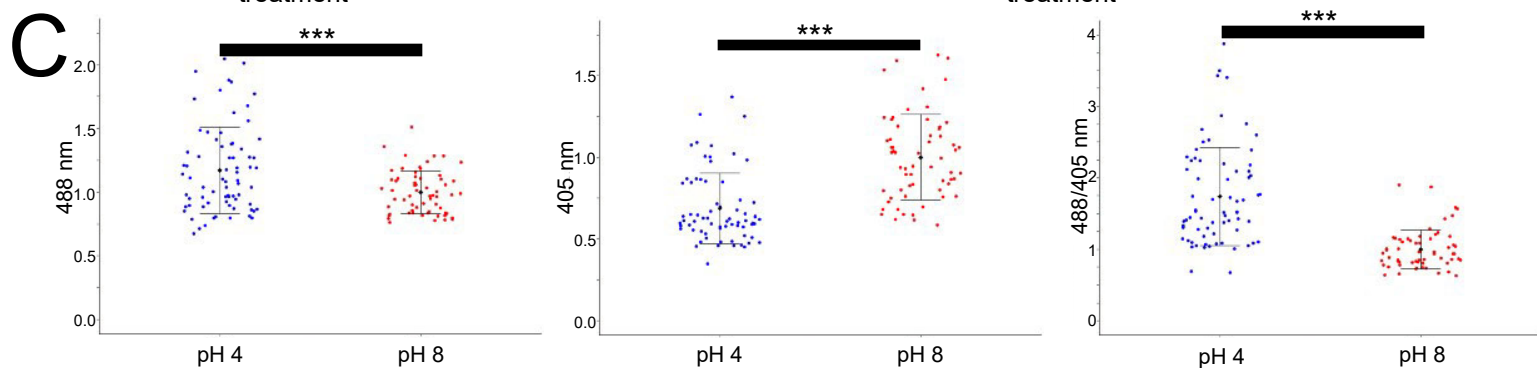

Supplement: Supplementary file 3 — Figure S3. pHlourin responds to environmental stimuli. (A) Tg(ubi:pHluorin2‐GPI) samples pre‐ and post‐treatment with the potassium ionophore Nigericin. 20× magnification. (B) Quantification of pH dysregulation caused by Nigericin treatment. (Left) pH independent fluorescence was unchanged but (Right) average embryo pH increased significantly. (C) Embryos soaked at pH 4 show higher global acidity than embryos at pH 8. (Left) 488 nm fluorescence increases in the acidic condition while (Middle) 405 nm fluorescence decreases. (Right) global fluorescence across the embryo is significantly increased in the acidic environment. (B) Representative data from n = 4 biological repeats with n = 45 embryos; normalized to pre‐treatment. (C) Data aggregated from n ≥ 4 biological repeats with n ≥ 62 embryos; normalized to pH 8. ***p < .001. [file DVDY-254-1068-s004.pdf]

405 nm

488 nm

Live

Fixed in 4% PFA

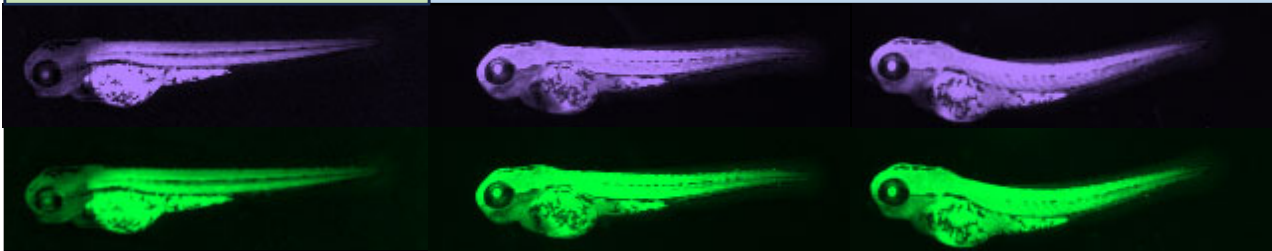

Supplement: Supplementary file 4 — Figure S4. 4% PFA fixation increases background. Representative images of Tg(ubi:pHluorin2‐GPI) embryos at 3 dpf pre‐ and post‐fixation. 2× magnification. n ≥ 3 biological replicates with n ≥ 10 embryos. [file DVDY-254-1068-s009.pdf]

**A**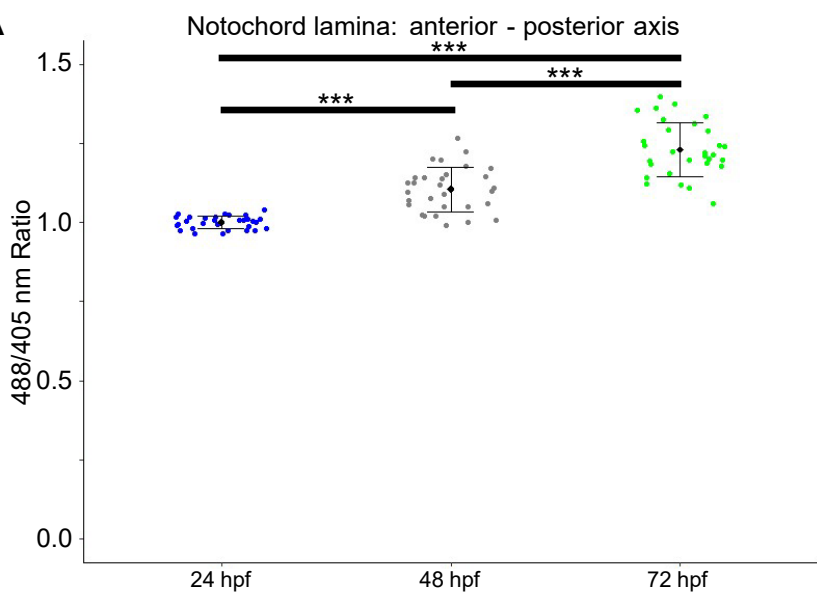**B**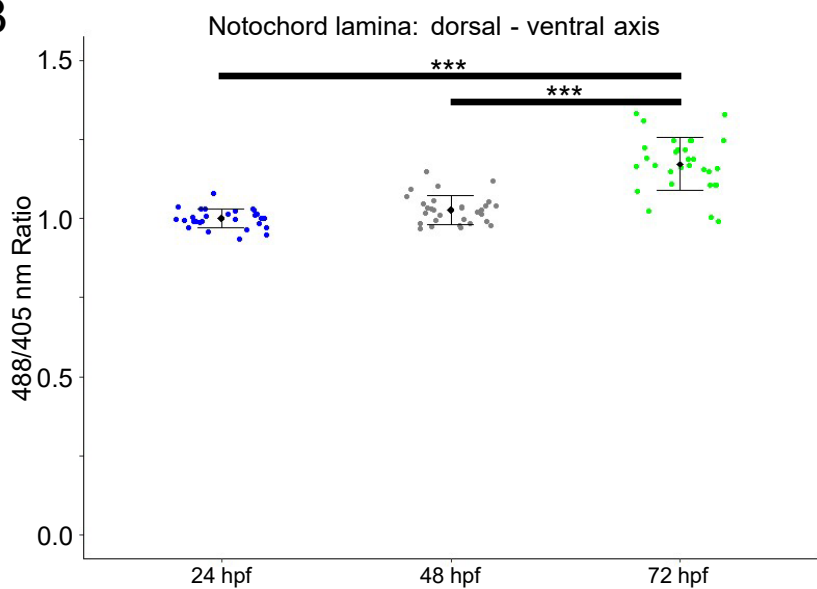**C**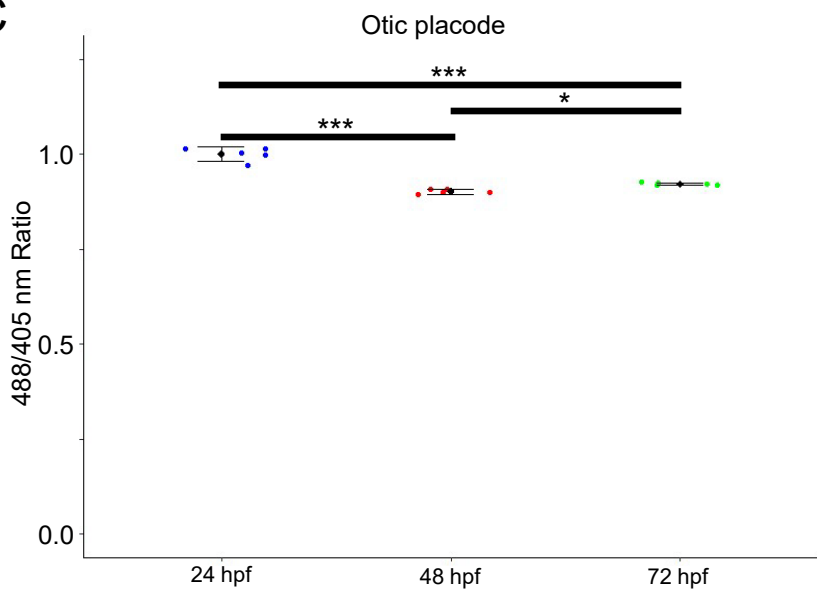

Supplement: Supplementary file 5 — Figure S5. Quantification of notochord lamina and otic placode. Acidification of the inter‐vacuole lamina along the (A) anterior–posterior axis and (B) dorsal‐ventral axis of the notochord increases from 24 to 72 hpf. (C) Acidification of the otic placode decreases from 24 to 72 hpf. (A) and (B) aggregate measurements of 5 different inter‐vacuole lamina from n = 6 embryos in n = 3 biological repeats. (C) n = 5 embryos from n = 3 biological replicates. (A–C) normalized to 24 hpf; * p < .05, ***p < .001. [file DVDY-254-1068-s003.pdf]

**A**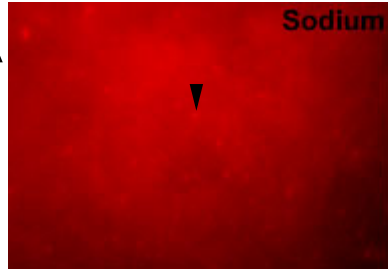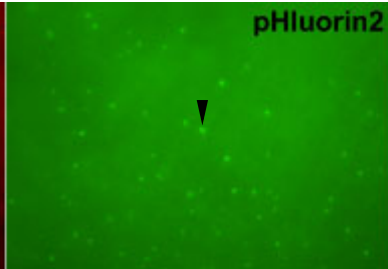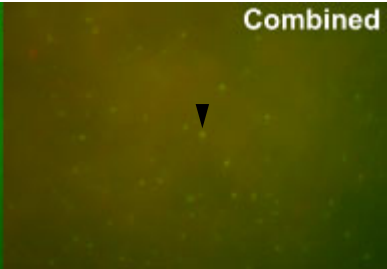**B**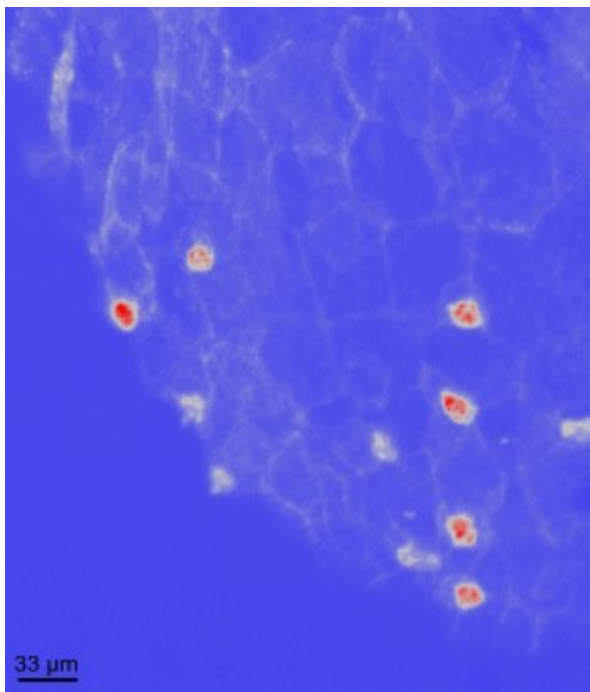

Supplement: Supplementary file 6 — Figure S6. pHlourin2‐GPI expression in ionocytes. (A) SBFI labeling of sodium rich H+‐ATPase‐rich (HR) ionocytes around the yolk sac coincides with high 488 nm signaling of pHlourin2‐GPI. Arrows indicate an ionocyte. (B) Magnified inset from Figure 3B. [file DVDY-254-1068-s002.pdf]

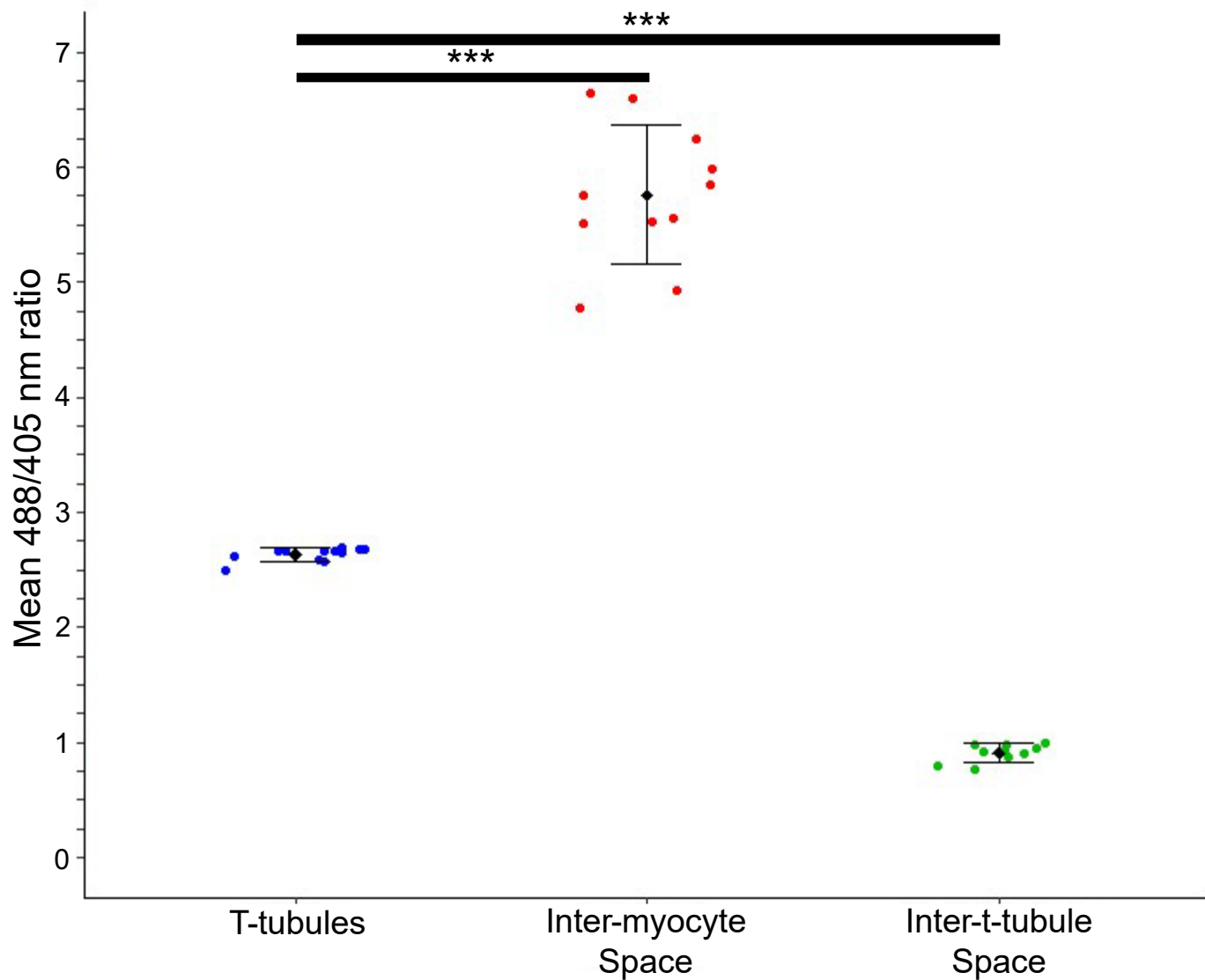

Supplement: Supplementary file 7 — Figure S7. Intermyocyte space is more proton‐rich than the T‐tubules. Quantification of 488/405 ratios at high resolution from Figure 4G shows that the intermyocyte space has a higher proton burden than the T‐tubules. Data aggregated from n = 3 embryos with each with 3–4 regions of interest for a minimum n ≥ 10; normalized to the inter‐t‐tubule space. ***p < .001. [file DVDY-254-1068-s008.pdf]

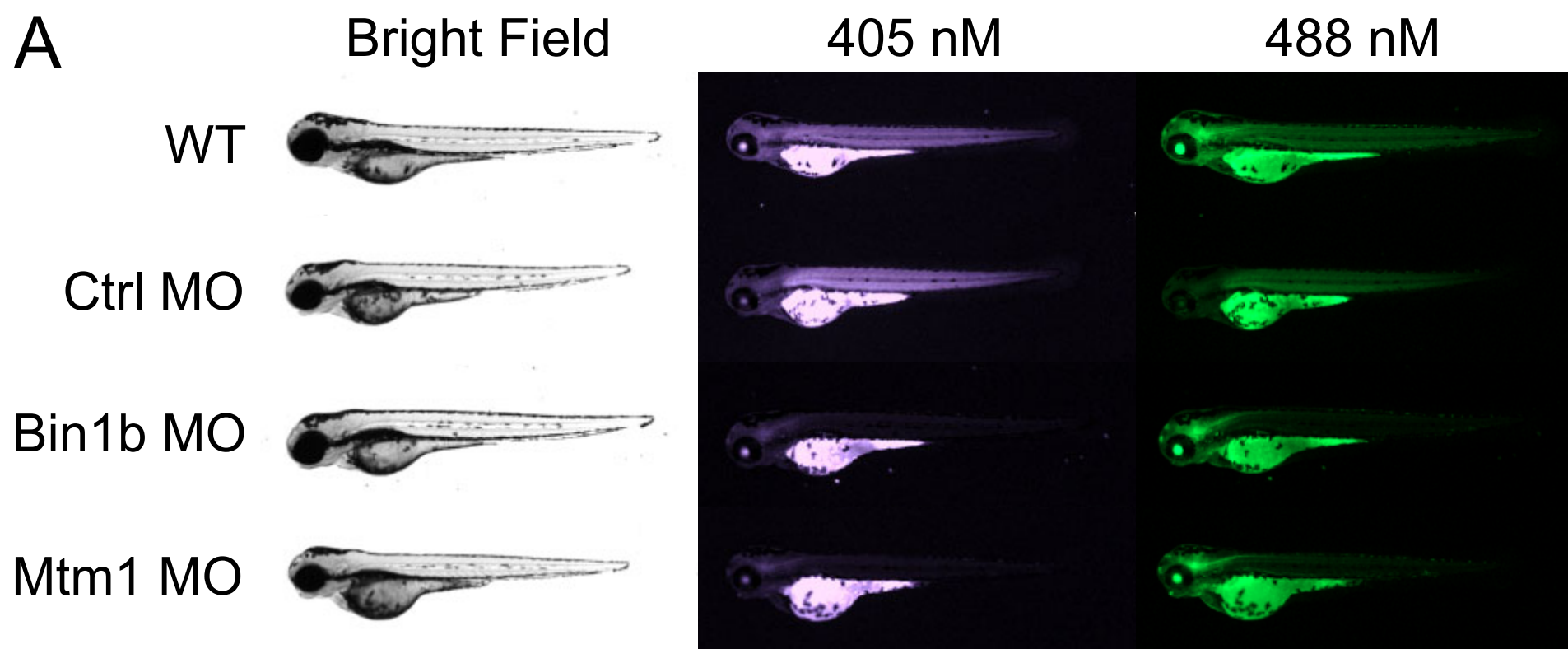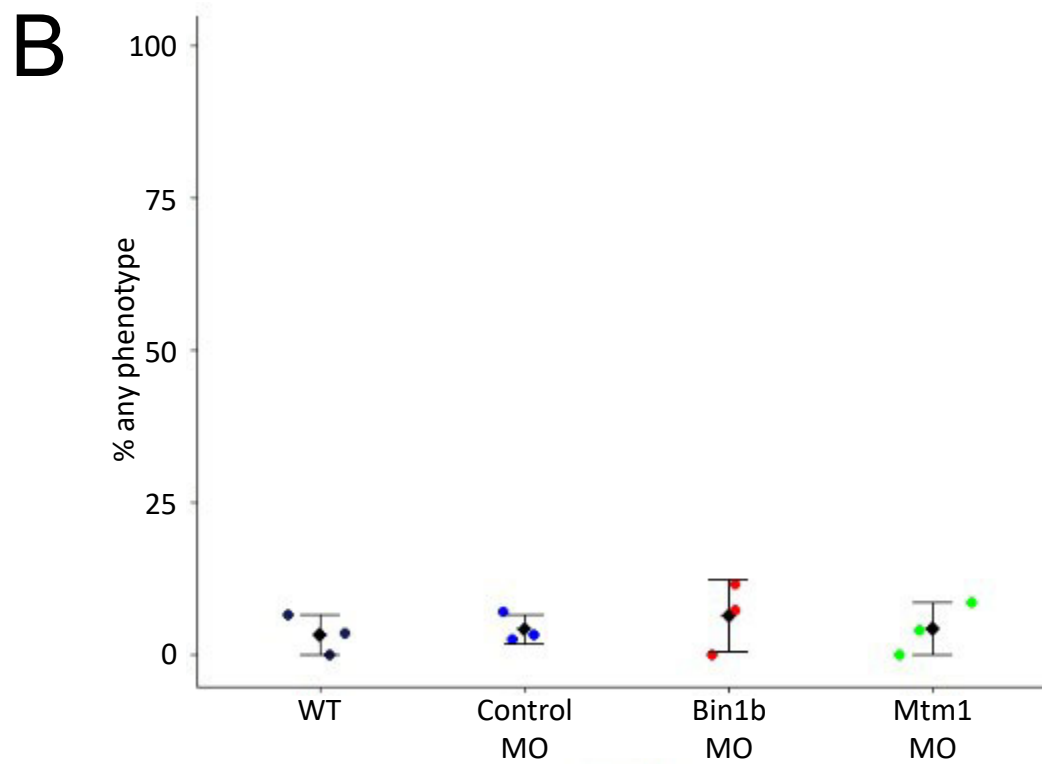

Supplement: Supplementary file 8 — Figure S8. Bin1b and Mtm1 decrease extracellular acidification of the embryonic myotome. (A) Whole animal photos of control MO, Bin1b MO, and Mtm1 MO in comparison to wild type controls. Knockdown of Bin1b and Mtm1 decrease expression of pHlourin2 at 72 hpf. (B) Quantification of all gross morphological changes in MO injected and WT groups. No repeatable gross malformations were observed, and no significant differences were observed between groups. (B) Averages of n = 3 biological replicates and a total of n ≥ 101 embryos from all groups combined. [file DVDY-254-1068-s005.pdf]
