# Supplementary material for: A novel transgenic reporter of extracellular acidification in zebrafish elucidates skeletal muscle T‐tubule pH regulation
Source: Dev Dyn. 2025 Jan 22;254(9):1068–79. doi: 10.1002/dvdy.770 (PMC12238821; doi:10.1002/dvdy.770)
Supplement: Supplementary file 9 — Supplementary File 1. [file DVDY-254-1068-s006.docx]

| pzTol2[Exp]-Ubi>{Phluorin2-GPI} | | | | |
| --- | --- | --- | --- | --- |
|  |  | 5' ITR |  |  |
|  | AmpiciIIin |  |  |  |
|  |  | 1 |  |  |
|  | 6000 |  |  |  |
|  | pUC ori |  |  |  |
|  |  | VB200601-1084rcb |  |  |
|  |  |  | 2000 |  |
|  |  | 7172 bp |  | Ubi |
|  | 3' ITR |  |  |  |
|  | SV40 late pA |  |  |  |
|  |  | 4000 |  |  |
|  | {Phluorin2-GPI} |  |  |  |
|  |  | Kozak |  |  |
| Backbone (5’ITR  underlined) | CAGAGGTGTAAAGTACTTGAGTAATTTTACTTGATTACTGTACTTAAGTATTATTTTTG GGGATTTTTACTTTACTTGAGTACAATTAAAAATCAATACTTTTACTTTTACTTAATTAC ATTTTTTTAGAAAAAAAAGTACTTTTTACTCCTTACAATTTTATTTACAGTCAAAAAGT ACTTATTTTTTGGAGATCACTTCATTCTATTTTCCCTTGCTATTACCAAACCAATTGAAT TGCGCTGATGCCCAGTTTAATTTAAATAGATCTCAACTTTGTATAGAAAAGTTG | | | |
| Zebrafish ubiquitin promoter | ACCAGCAAAGTTCTAGAATTTGTCGAAACATTTATGTTATATATTTCCTGAAAAAAATT CTGAGTAAGTTCTTAAGTGTTATTGCCAGCAACATAAACAACAGACGGCAAAATGAA TAAATGATAACAAAGCAGTAGGCTTAAATAAACCTAATTTTTATAGGCTGTTCTCTACA ACCCTCAAACAGTGATTAGTTTTGTACTTATAAACTTGCCCTTTCATTCATATTTCAAG AAAATTGGTTCAGAAGATCTGGATATTCTAGCAGTTGTTCAAGCTCATGGAGGGATC AGTGACCTGATTCCACAATGACTAGGCCTAATCCAGAAATTAGATGACTGTCAACATA AAAAGGCACAGCACTCACTAGCTGCCCTATATATTTTATTATATTTTACATATATTATTTT ATTTATTTAGCTCTGAGTGCTGTACTTTCTGGTTAAAGAAAACTGCTTACAACAGCTA ACCTGTACTACCTCAGGCTCAGGGAATTTGGAACAGGTTTGTCTGGTTTGTTTCTTT AACCATGCATGCTTGTTTTCAACTATGGCAACACAGTCACATGGGACATTACAGAAA TGATTTGTCGATGACATGCGACTTTTCTTTAATAAAGCGCAAAGATCCCAAAAAGCA AACTTTTAACAAAAATCATATAATTATATTTTCAATCCAGCTTTGTAGCAACTTTGTGCT GCTGTTCACTCAGCAACAGATAGTCAGTATAAGGTCAGTGTGTCTCAAAGCAGTGCC ATCTGTTTCACACATTGCGTTCTATATATAAGTGTGCTGGTTGACACGACACTGTATAA GGCCTAGGCTAAAACACAAACAATGTAGAATGACACTGTGTTTTTTTTGTAAACAAA TGTTGTTTTTGGTTAAACATCTTTGTGAAAACATCCTCCTGTCATGTATTTGCTATATTC AAATGTTAAACCCGTGCAGAATAGAACATATACAAAAAAAAACAACACAACACATTT TTAAACATTATTAAATATCAAGTATTGCTGGCAGTTCTGTTTCTGTTTTACAGTACCCT TTGCCACAGTTCTCCGCTTTTCCTGGTCCAGATTCCACAAGTCTGATTCACCAATAGC AAAGCGAATAAACAACCAAAGCAGCCAATCACTGCTTGTAGACTGTCCTGCGAGAC CGGCCCATTCCAGCACATTCTGGAAACTTCCTTTATATGATAATTATAAATACATTTAA ATTATTGATACAAAACATGTAATTCCTAGAACATAACCATAGCAATCATTAGTTTTCAG  GGTAATTATGTATTTTTAGGATTTGACTGCGGAAAGATCTGGTCATGTGACGTCTCAT GAACGTCACGGCCCTGGGTTTCTATAAATACAGTAGGACTCTCGACCATCGGCAGAT | | | |


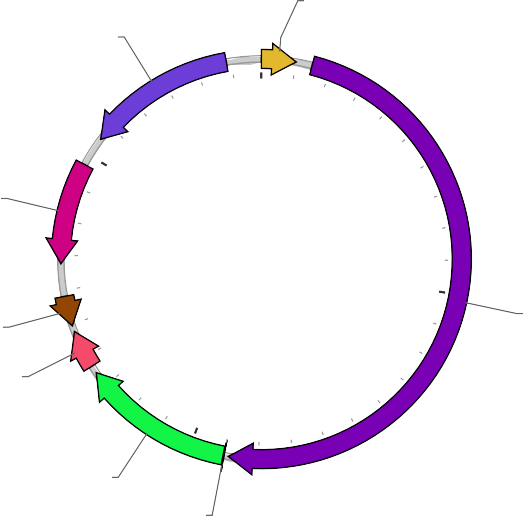


|  | TTTTCGAAGAAGAAGATCAGTTTCAGGAGCCGTACTGTTCCGTTTCAACGCAAATAT TAACGGTAAGAGCGAATTTCCTAGTTTGTTTTCATGCCATTCTTTAAAACCATAGCGT ATTACTTTAATTATAGTAAACTTTCGCTTTCTTTATTACAAGAGACGTTTTGTGTTGATT CTCCGCGGACATTTTCGGTCAGACAATCAGAAAATGACCGCGGAGGACCAGTAACT TGCATTACACGTAAGTTAAATCTTCGTGTATTAAAATGGTTAGGTTGTTAACGTCAAAT AGGTTACCGTGTTTGCGTGTGATCAGGTTGGTTTTGTTAGATTTTTGTCAGTATTTTT AATTTATTTGTTTTAGTTTATTTATTTTTTTTGCTGAATCATAGTTTGTGAACAAAGAAC CCGGATGTTACATACAGTACAGCCGCCATGTTACAGAGAGTTATAACTTAATCATTTTA AAAATAATTTTGCCTTACTTTTAGTTTGTCATGTTGAGAAATGAGGAAATGTTAAAAT GAGGAAATATCCAATTAATTTAATATATCAAAATAATCCATGATTACAATGCACTGAACT GGAGAAAATTAAGATGTTTTCTAGTGTCATGAAACAAATGTAAGAGATGTACATTGTA GATGTTTTATGTCAAGAATTGGCTAGTTGATGCAGCATACTGGCGATACTCAGTTGTA ATAACAGTAACGTTACATGTTAATAGACTACTGAGTATGCTGTTCTGTCTATGTATGCT CTGTAAGCTACGAGAAGGACTTTTTTAAACAGTAAAGGGTGCAATATTTTTACAAAT TGAATTAAATAAAGGCTGTCTATTAAGTAATATGCTTGATATTTTTCTTACTTGATCGGA AATAAGAAAAAATATAAACGTTGTTGCTCTAAAAATCCTAGTTCAGTTTAGCCAACCA CAAATACCTTTTTGTTCCTCCAACAGTTTTTTTTTCTTCTCTATAATATTTGGCAGTCTA TAGTACTCCAAATGTTTCCCCACAGTCTAACTAATTGGTACAGCCAAAATCATGACAC TTATTGCAATAATAATTTTGGTTCATTGGCATTGTTGATAGCCTGTGCCACTAATATGG TCGATTGATCATGCTTCAGGAAGAAAACTATATTGTTTGATGTAAGATTATTAAATCTT CACCTGCTTCCATTACAAACTATTCCCATCTTATTGAATTCTGGTATGTCTTAAAGGAT TAGTTCACTTCCCAAATCAAAATTTACTTAGCCTTTTTTCATCCATGATCCCTTTTTTTC ATCATTAATGAAGAAATTGTTTTTGAAAAAGTTTCAAGATTTTTTTCTCTATATTGTGG AGCTTGTTAGTTTAAAATTCCAAAATGCAATATGTGGCTTCAAATGGTTCTAAATGATC CCAGTCAAGGAATAACAGTCTTATCTAATGAAACCATTAGACCTTTAAAAATAAAAAA AATAAAAGTATTTATTTTTAAATGACTGAGTGATTAAGTTGAATTTCAGCGTTTCCTTA CTGTGTAGAAGTCCTTCCTTACTGGCCCCACCCTTTGGTTCTCTGCCAATCTGCTACC TAATGTAATGTTGTGGAACATTATTATTCTTTATTTCTTAATTTTTTATTTTTTATTTTAA AACAATGTAAACTGCACAGATGTGCAGTTTGTTTAAAATGGCCAATGCTTTGGAAAT GCATGACATAATTAGATTTCATGATGCACAAAGCCAAATCTCAGAGCTTGTGCAAAAT GAGCTATCATTTCACTAGGTAAGACCCTAAATTTTCATATAGGATCATTTGGACAATTT TGCTGCAGGTAAAATGCATTCTATAGTCCACTGTCAGCCATTGTTTTGGATAGTATTTA TTTTTCTCTACAAGTATAGTCAATAGTTTTCTATTATTTTAAAGGTTTGTAACATTTAAG GGTGACCAAATGCAAAGTAAAATTTCATTTTCGGGTGAACTATCTCGTTTAACATGG GAGAAGTGCAAAACATACATTATTGGCTAGAACATTGTAGTATTTTTTAAATGGAAAT  GTGTGATTGCTAATCTTACTTTGAATTTGTTTACAG |
| --- | --- |
| Linker | CAAGTTTGTACAAAAAAGCAGGCTGCCACC |
| Kozak  sequence | GCCACC |
| pHLuorin2 | ATGGCTCAGCGGATGACAACACAGCTGCTGCTCCTTCTAGTGTGGGTGGCTGTAGT AGGGGAGGCTCAGACAAGGATTGCAATGGTGAGCAAGGGCGAGGAGCTGTTCAC CGGGGTGGTGCCCATCCTGGTCGAGCTGGACGGCGACGTAAACGGCCACAAGTTC AGCGTGTCCGGCGAGGGCGAGGGCGATGCCACCTACGGCAAGCTGACCCTGAAGT  TCATCTGCACCACCGGCAAGCTGCCCGTGCCCTGGCCCACCCTCGTGACCACCCTG |

|  | AGCTACGGCGTGCAGTGCTTCAGCCGCTACCCCGACCACATGAAGCAGCACGACTT CTTCAAGTCCGCCATGCCCGAAGGCTACGTCCAGGAGCGCACCATCTTCTTCAAGG ACGACGGCAACTACAAGACCCGCGCCGAGGTGAAGTTCGAGGGCGACACCCTGGT GAACCGCATCGAGCTGAAGGGCATCGACTTCAAGGAGGACGGCAACATCCTGGGG CACAAGCTGGAGTACAACTACAACGAGCACCTGGTGTACATCATGGCCGACAAGCA GAAGAACGGCACCAAGGCCATCTTCCAGGTGCACCACAACATCGAGGACGGCAGC GTGCAGCTCGCCGACCACTACCAGCAGAACACCCCCATCGGCGACGGCCCCGTGCT GCTGCCCGACAACCACTACCTGCACACCCAGTCCGCCCTGAGCAAAGACCCCAACG AGAAGCGCGATCACATGGTCCTGCTGGAGTTCGTGACCGCCGCCGGGATCACTCAC  GGCATGGACGAGCTGTACAAG |
| --- | --- |
| Linker | GCTGCAGCC |
| GPI  (from the  human folate receptor) | ATGAGTGGGGCTGGGCCCTGGGCAGCCTGGCCTTTCCTGCTTAGCCTGGCCCTAAT GCTGCTGTGGCTGCTCAGCTGA |
| Backbone (3’ITR  underlined) | ACCCAGCTTTCTTGTACAAAGTGGTGATGGCCGGCCGCTTCGAGCAGACATGATAA GATACATTGATGAGTTTGGACAAACCACAACTAGAATGCAGTGAAAAAAATGCTTTA TTTGTGAAATTTGTGATGCTATTGCTTTATTTGTAACCATTATAAGCTGCAATAAACAA GTTAACAACAACAATTGCATTCATTTTATGTTTCAGGTTCAGGGGGAGGTGTGGGA GGTTTTTTAAAGCAAGTAAAACCTCTACAAATGTGGTAGATATCAAGCTTAAACAAG AATCTCTAGTTTTCTTTCTTGCTTTTACTTTTACTTCCTTAATACTCAAGTACAATTTTA ATGGAGTACTTTTTTACTTTTACTCAAGTAAGATTCTAGCCAGATACTTTTACTTTTAA TTGAGTAAAATTTTCCCTAAGTACTTGTACTTTCACTTGAGTAAAATTTTTGAGTACTT TTTACACCTCTGGGCGCTCTTCCGCTTCCTCGCTCACTGACTCGCTGCGCTCGGTCG TTCGGCTGCGGCGAGCGGTATCAGCTCACTCAAAGGCGGTAATACGGTTATCCACA GAATCAGGGGATAACGCAGGAAAGAACATGTGAGCAAAAGGCCAGCAAAAGGCC AGGAACCGTAAAAAGGCCGCGTTGCTGGCGTTTTTCCATAGGCTCCGCCCCCCTGA CGAGCATCACAAAAATCGACGCTCAAGTCAGAGGTGGCGAAACCCGACAGGACTAT AAAGATACCAGGCGTTTCCCCCTGGAAGCTCCCTCGTGCGCTCTCCTGTTCCGACCC TGCCGCTTACCGGATACCTGTCCGCCTTTCTCCCTTCGGGAAGCGTGGCGCTTTCTC ATAGCTCACGCTGTAGGTATCTCAGTTCGGTGTAGGTCGTTCGCTCCAAGCTGGGCT GTGTGCACGAACCCCCCGTTCAGCCCGACCGCTGCGCCTTATCCGGTAACTATCGTC TTGAGTCCAACCCGGTAAGACACGACTTATCGCCACTGGCAGCAGCCACTGGTAAC AGGATTAGCAGAGCGAGGTATGTAGGCGGTGCTACAGAGTTCTTGAAGTGGTGGC CTAACTACGGCTACACTAGAAGAACAGTATTTGGTATCTGCGCTCTGCTGAAGCCAG TTACCTTCGGAAAAAGAGTTGGTAGCTCTTGATCCGGCAAACAAACCACCGCTGGT AGCGGTGGTTTTTTTGTTTGCAAGCAGCAGATTACGCGCAGAAAAAAAGGATCTCA AGAAGATCCTTTGATCTTTTCTACGGGGTCTGACGCTCAGTGGAACGAAAACTCAC GTTAAGGGATTTTGGTCATGAGATTATCAAAAAGGATCTTCACCTAGATCCTTTTAAA TTAAAAATGAAGTTTTAAATCAATCTAAAGTATATATGAGTAAACTTGGTCTGACAGTT ACCAATGCTTAATCAGTGAGGCACCTATCTCAGCGATCTGTCTATTTCGTTCATCCATA GTTGCCTGACTCCCCGTCGTGTAGATAACTACGATACGGGAGGGCTTACCATCTGGC  CCCAGTGCTGCAATGATACCGCGAGATCCACGCTCACCGGCTCCAGATTTATCAGCA ATAAACCAGCCAGCCGGAAGGGCCGAGCGCAGAAGTGGTCCTGCAACTTTATCCG |

|  | CCTCCATCCAGTCTATTAATTGTTGCCGGGAAGCTAGAGTAAGTAGTTCGCCAGTTAA TAGTTTGCGCAACGTTGTTGCCATTGCTACAGGCATCGTGGTGTCACGCTCGTCGTT TGGTATGGCTTCATTCAGCTCCGGTTCCCAACGATCAAGGCGAGTTACATGATCCCC CATGTTGTGCAAAAAAGCGGTTAGCTCCTTCGGTCCTCCGATCGTTGTCAGAAGTAA GTTGGCCGCAGTGTTATCACTCATGGTTATGGCAGCACTGCATAATTCTCTTACTGTC ATGCCATCCGTAAGATGCTTTTCTGTGACTGGTGAGTACTCAACCAAGTCATTCTGAG AATAGTGTATGCGGCGACCGAGTTGCTCTTGCCCGGCGTCAATACGGGATAATACCG CGCCACATAGCAGAACTTTAAAAGTGCTCATCATTGGAAAACGTTCTTCGGGGCGA AAACTCTCAAGGATCTTACCGCTGTTGAGATCCAGTTCGATGTAACCCACTCGTGCA CCCAACTGATCTTCAGCATCTTTTACTTTCACCAGCGTTTCTGGGTGAGCAAAAACA GGAAGGCAAAATGCCGCAAAAAAGGGAATAAGGGCGACACGGAAATGTTGAATAC TCATACTCTTCCTTTTTCAATATTATTGAAGCATTTATCAGGGTTATTGTCTCATGAGCG GATACATATTTGAATGTATTTAGAAAAATAAACAAATAGGGGTTCCGCGCACATTTCC CCGAAAAGTGCCACCTGACGTCTAAGAAACCATTATTATCATGACATTAACCTATAAA  AATAGGCGTATCACGAGGCCCTTTCGTC |
| --- | --- |

| pT7[mRNA]-{Tol2} | |
| --- | --- |
| T7 promoter  1  AmpiciIIin  {Tol2}  3000 VB220830-1237vmx 1000  4030 bp  2000  pUC ori  poly(A)  SapI  AscI BsiWI | |
| T7 promoter | TAATACGACTCACTATAGG |
| Tol2 | ATGGAGGAAGTATGTGATTCATCAGCAGCTGCGAGCAGCACAGTCCAAAATCAGCC ACAGGATCAAGAGCACCCGTGGCCGTATCTTCGCGAATTCTTTTCTTTAAGTGGTGT AAATAAAGATTCATTCAAGATGAAATGTGTCCTCTGTCTCCCGCTTAATAAAGAAATAT CGGCCTTCAAAAGTTCGCCATCAAACCTAAGGAAGCATATTGAGAGAATGCACCCA AATTACCTCAAAAACTACTCTAAATTGACAGCACAGAAGAGAAAGATCGGGACCTCC ACCCATGCTTCCAGCAGTAAGCAACTGAAAGTTGACTCAGTTTTCCCAGTCAAACAT GTGTCTCCAGTCACTGTGAACAAAGCTATATTAAGGTACATCATTCAAGGACTTCATC CTTTCAGCACTGTTGATCTGCCATCATTTAAAGAGCTGATTAGTACACTGCAGCCTGG CATTTCTGTCATTACAAGGCCTACTTTACGCTCCAAGATAGCTGAAGCTGCTCTGATC ATGAAACAGAAAGTGACTGCTGCCATGAGTGAAGTTGAATGGATTGCAACCACAAC GGATTGTTGGACTGCACGTAGAAAGTCATTCATTGGTGTAACTGCTCACTGGATCAA CCCTGGAAGTCTTGAAAGACATTCCGCTGCACTTGCCTGCAAAAGATTAATGGGCTC TCATACTTTTGAGGTACTGGCCAGTGCCATGAATGATATCCACTCAGAGTATGAAATA CGTGACAAGGTTGTTTGCACAACCACAGACAGTGGTTCCAACTTTATGAAGGCTTT CAGAGTTTTTGGTGTGGAAAACAATGATATCGAGACTGAGGCAAGAAGGTGTGAA AGTGATGACACTGATTCTGAAGGCTGTGGTGAGGGAAGTGATGGTGTGGAATTCCA AGATGCCTCACGAGTCCTGGACCAAGACGATGGCTTCGAATTCCAGCTACCAAAAC ATCAAAAGTGTGCCTGTCACTTACTTAACCTAGTCTCAAGCGTTGATGCCCAAAAAG CTCTCTCAAATGAGCACTACAAGAAACTCTACAGATCTGTCTTTGGCAAATGCCAAG CTTTATGGAATAAAAGCAGCCGATCGGCTCTAGCAGCTGAAGCTGTTGAATCAGAAA GCCGGCTTCAGCTTTTAAGGCCAAACCAAACGCGGTGGAATTCAACTTTTATGGCT GTTGACAGAATTCTTCAAATTTGCAAAGAAGCAGGAGAAGGCGCACTTCGGAATAT ATGCACCTCTCTTGAGGTTCCAATGTTTAATCCAGCAGAAATGCTGTTCTTGACAGA GTGGGCCAACACAATGCGTCCAGTTGCAAAAGTACTCGACATCTTGCAAGCGGAAA CGAATACACAGCTGGGGTGGCTGCTGCCTAGTGTCCATCAGTTAAGCTTGAAACTTC AGCGACTCCACCATTCTCTCAGGTACTGTGACCCACTTGTGGATGCCCTACAACAAG  GAATCCAAACACGATTCAAGCATATGTTTGAAGATCCTGAGATCATAGCAGCTGCCAT |


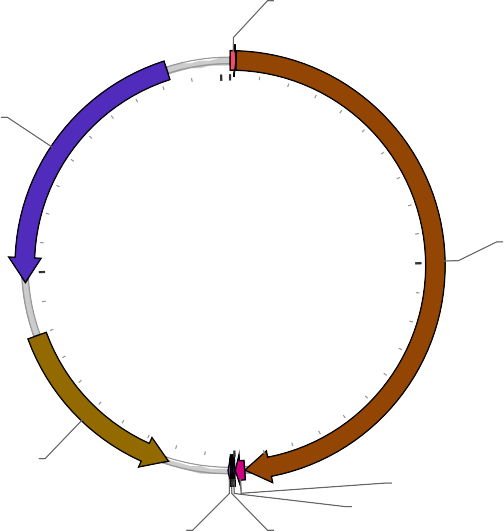


|  | CCTTCTCCCTAAATTTCGGACCTCTTGGACAAATGATGAAACCATCATAAAACGAGG CATGGACTACATCAGAGTGCATCTGGAGCCTTTGGACCACAAGAAGGAATTGGCCA ACAGTTCATCTGATGATGAAGATTTTTTCGCTTCTTTGAAACCGACAACACATGAAG CCAGCAAAGAGTTGGATGGATATCTGGCCTGTGTTTCAGACACCAGGGAGTCTCTG CTCACGTTTCCTGCTATTTGCAGCCTCTCTATCAAGACTAATACACCTCTTCCCGCATC GGCTGCCTGTGAGAGGCTTTTCAGCACTGCAGGATTGCTTTTCAGCCCCAAAAGAG CTAGGCTTGACACTAACAATTTTGAGAATCAGCTTCTACTGAAGTTAAATCTGAGGTT  TTACAACTTTGAGTAG |
| --- | --- |
| Backbone | AAAAAAAAAAAAAAAAAAAAAAAAAAAAAATGAAGAGCCGTACGGGCGCGCCTAG GCGCGATTCCGCTTCCTCGCTCACTGACTCGCTGCGCTCGGTCGTTCGGCTGCGGC GAGCGGTATCAGCTCACTCAAAGGCGGTAATACGGTTATCCACAGAATCAGGGGAT AACGCAGGAAAGAACATGTGAGCAAAAGGCCAGCAAAAGGCCAGGAACCGTAAA AAGGCCGCGTTGCTGGCGTTTTTCCATAGGCTCCGCCCCCCTGACGAGCATCACAA AAATCGACGCTCAAGTCAGAGGTGGCGAAACCCGACAGGACTATAAAGATACCAGG CGTTTCCCCCTGGAAGCTCCCTCGTGCGCTCTCCTGTTCCGACCCTGCCGCTTACCG GATACCTGTCCGCCTTTCTCCCTTCGGGAAGCGTGGCGCTTTCTCATAGCTCACGCT GTAGGTATCTCAGTTCGGTGTAGGTCGTTCGCTCCAAGCTGGGCTGTGTGCACGAA CCCCCCGTTCAGCCCGACCGCTGCGCCTTATCCGGTAACTATCGTCTTGAGTCCAAC CCGGTAAGACACGACTTATCGCCACTGGCAGCAGCCACTGGTAACAGGATTAGCAG AGCGAGGTATGTAGGCGGTGCTACAGAGTTCTTGAAGTGGTGGCCTAACTACGGCT ACACTAGAAGAACAGTATTTGGTATCTGCGCTCTGCTGAAGCCAGTTACCTTCGGAA AAAGAGTTGGTAGCTCTTGATCCGGCAAACAAACCACCGCTGGTAGCGGTGGTTTT TTTGTTTGCAAGCAGCAGATTACGCGCAGAAAAAAAGGATCTCAAGAAGATCCTTT GATCTTTTCTACGGGGTCTGACGCTCAGTGGAACGAAAACTCACGTTAAGGGATTT TGGTCATGAGATTATCAAAAAGGATCTTCACCTAGATCCTTTTAAATTAAAAATGAAG TTTTAAATCAATCTAAAGTATATATGAGTAAACTTGGTCTGACAGTTACCAATGCTTAA TCAGTGAGGCACCTATCTCAGCGATCTGTCTATTTCGTTCATCCATAGTTGCCTGACT CCCCGTCGTGTAGATAACTACGATACGGGAGGGCTTACCATCTGGCCCCAGTGCTGC AATGATACCGCGAGATCCACGCTCACCGGCTCCAGATTTATCAGCAATAAACCAGCC AGCCGGAAGGGCCGAGCGCAGAAGTGGTCCTGCAACTTTATCCGCCTCCATCCAGT CTATTAATTGTTGCCGGGAAGCTAGAGTAAGTAGTTCGCCAGTTAATAGTTTGCGCA ACGTTGTTGCCATTGCTACAGGCATCGTGGTGTCACGCTCGTCGTTTGGTATGGCTT CATTCAGCTCCGGTTCCCAACGATCAAGGCGAGTTACATGATCCCCCATGTTGTGCA AAAAAGCGGTTAGCTCCTTCGGTCCTCCGATCGTTGTCAGAAGTAAGTTGGCCGCA GTGTTATCACTCATGGTTATGGCAGCACTGCATAATTCTCTTACTGTCATGCCATCCGT AAGATGCTTTTCTGTGACTGGTGAGTACTCAACCAAGTCATTCTGAGAATAGTGTAT GCGGCGACCGAGTTGCTCTTGCCCGGCGTCAATACGGGATAATACCGCGCCACATA GCAGAACTTTAAAAGTGCTCATCATTGGAAAACGTTCTTCGGGGCGAAAACTCTCA AGGATCTTACCGCTGTTGAGATCCAGTTCGATGTAACCCACTCGTGCACCCAACTGA TCTTCAGCATCTTTTACTTTCACCAGCGTTTCTGGGTGAGCAAAAACAGGAAGGCA AAATGCCGCAAAAAAGGGAATAAGGGCGACACGGAAATGTTGAATACTCATACTCT TCCTTTTTCAATATTATTGAAGCATTTATCAGGGTTATTGTCTCATGAGCGGATACATAT  TTGAATGTATTTAGAAAAATAAACAAATAGGGGTTCCGCGCACATTTCCCCGAAAAG |

|  | TGCCACCTGACGTCTAAGAAACCATTATTATCATGACATTAACCTATAAAAATAGGCGT  ATCACGAGGCCCTTTCGTC |
| --- | --- |
